# An evaluation of data quality in Canada’s Continuing Care Reporting System (CCRS): secondary analyses of Ontario data submitted between 1996 and 2011

**DOI:** 10.1186/1472-6947-13-27

**Published:** 2013-02-26

**Authors:** John P Hirdes, Jeff W Poss, Hilary Caldarelli, Brant E Fries, John N Morris, Gary F Teare, Kristen Reidel, Norma Jutan

**Affiliations:** 1 School of Public Health and Health Systems, University of Waterloo, 200 University Avenue West, N2L 3G1, Waterloo, ON, Canada; 2 Institute of Gerontology, University of Michigan, 300 North Ingalls, 48109, Ann Arbor, MI, USA; 3 Ann Arbor VA Health Care Center, Geriatrics Research, Education and Clinical Center, 2215 Fuller Road, 48105, Ann Arbor, MI, USA; 4 Institute for Aging Research, 1200 Centre Street, 02131, Boston, MA, USA; 5 Saskatchewan Health Quality Council, Atrium Building, Innovation Place, 241 – 111 Research Drive, SK S7N 3R2, Saskatoon, Canada; 6 IMS Brogan, Montreal 16720 Route Transcanadienne Kirkland, H9H 5M3, Quebec, Canada; 7 Canadian Institute for Health Information, Home and Continuing Care, Ottawa, 495 Richmond Road, Suite 600, K2A 4H6, Ottawa, ON, Canada

**Keywords:** interRAI, RAI 2.0, Minimum Data Set, MDS, Assessment, Quality, Funding

## Abstract

**Background:**

Evidence informed decision making in health policy development and clinical practice depends on the availability of valid and reliable data. The introduction of interRAI assessment systems in many countries has provided valuable new information that can be used to support case mix based payment systems, quality monitoring, outcome measurement and care planning. The Continuing Care Reporting System (CCRS) managed by the Canadian Institute for Health Information has served as a data repository supporting national implementation of the Resident Assessment Instrument (RAI 2.0) in Canada for more than 15 years. The present paper aims to evaluate data quality for the CCRS using an approach that may be generalizable to comparable data holdings internationally.

**Methods:**

Data from the RAI 2.0 implementation in Complex Continuing Care (CCC) hospitals/units and Long Term Care (LTC) homes in Ontario were analyzed using various statistical techniques that provide evidence for trends in validity, reliability, and population attributes. Time series comparisons included evaluations of scale reliability, patterns of associations between items and scales that provide evidence about convergent validity, and measures of changes in population characteristics over time.

**Results:**

Data quality with respect to reliability, validity, completeness and freedom from logical coding errors was consistently high for the CCRS in both CCC and LTC settings. The addition of logic checks further improved data quality in both settings. The only notable change of concern was a substantial inflation in the percentage of long term care home residents qualifying for the Special Rehabilitation level of the Resource Utilization Groups (RUG-III) case mix system after the adoption of that system as part of the payment system for LTC.

**Conclusions:**

The CCRS provides a robust, high quality data source that may be used to inform policy, clinical practice and service delivery in Ontario. Only one area of concern was noted, and the statistical techniques employed here may be readily used to target organizations with data quality problems in that (or any other) area. There was also evidence that data quality was good in both CCC and LTC settings from the outset of implementation, meaning data may be used from the entire time series. The methods employed here may continue to be used to monitor data quality in this province over time and they provide a benchmark for comparisons with other jurisdictions implementing the RAI 2.0 in similar populations.

## Background

Evidence-informed decision-making is an essential ingredient of any strategy that aims to deal with the complex challenges posed by population changes, limited resources, advancements in technology, and changing public expectations. High quality information systems are a foundation on which to build evidence to inform decisions. The availability of standardized, representative, comprehensive, reliable and valid data is a precondition to formulating evidence to respond to those challenges. Therefore, an essential step in introducing large-scale data systems aimed at providing such evidence is an evaluation of the quality of those data [1-3].

There are many potential threats to the quality of data in any large health information system. Unless the psychometric properties of the data have been evaluated systematically, one cannot have confidence the information on which decisions are made is inherently reliable or valid, even if the system is implemented and maintained in optimal conditions. Such information systems should perform not only in ideal conditions, but also when faced with the rigors of day-to-day use. Examples of factors that can undermine the quality of data in “real-world” situations include: poor training and lack of on-going education; lack of staff expertise; inadequate “buy-in” by staff; systematic biases in reporting due to financial incentives or avoidance of negative consequences of unfavorable findings; temporary coding problems with the introduction of new systems; declining attention and underfunding; lack of feedback to users; and poor data collection or coding strategies.

### The Resident Assessment Instrument 2.0 and Continuing Care Reporting System (CCRS)

The Resident Assessment Instrument 2.0 (RAI 2.0) is a comprehensive assessment system that is at different stages of implementation in two care settings in eight Canadian provinces/territories [4]: long term care facilities (i.e., nursing homes that tend to serve a long-stay, medically stable population with substantial impairments in cognition or physical function) and complex continuing care hospitals/units (post-acute hospital settings that serve medically unstable persons with complex health conditions and functional impairments with stays typically lasting less than 90 days).

The instrument was originally developed by United States (US) researchers [5] after passage of a US law aimed at improving the quality of nursing home care [6]. Since then the system has been maintained and improved by interRAI (http://www.interrai.org), a 32-country network of clinicians and researchers focused on the implementation, application and continuing refinement of a suite of compatible instruments including the RAI 2.0 [7,8]. interRAI’s goal is to develop these assessment systems to comprise an integrated health information system linking multiple sectors of health and social services for the elderly and other vulnerable populations [9,10].

The RAI 2.0 is composed of three main components. First, the Minimum Data Set (MDS) data collection form, which includes about 440 items covering domains such as cognition, communication, mood and behaviour, psychosocial well-being, physical functioning, continence, health conditions, nutrition, activities, medication, treatments, procedures, and discharge potential. Second, a corresponding manual with item-by-item descriptions outlining the definitions, intent, assessment process, and coding rules. Third, Clinical Assessment Protocols (CAPs), which support development of care plans in 22 clinical areas [11]. The RAI 2.0 is designed as a comprehensive assessment to be used as part of normal clinical practice, and assessments are intended to be done by trained health professionals working as part of a multidisciplinary team. In addition to care planning [12], other applications include case-mix based funding [13], quality indicators [14,15], and outcome measurement [16]. These multiple applications for multiple audiences have meant that many stakeholders in continuing care have begun to look to these data as an important source of information for a variety of decisions. For example, the Resource Utilization Groups (RUG-III) case-mix algorithm [13] is now used in the funding formula for both complex continuing care (CCC) hospitals/units [17] and long term care (LTC) homes in Ontario [18]. In addition, Health Quality Ontario (http://www.hqontario.ca) reports publicly on CCC and LTC performance using quality indicators from the RAI 2.0 [15,19]. Some of these indicators are part of formal accountability agreements between health care organizations and government agencies at the local and provincial levels that link performance expectations to funding.

The Continuing Care Reporting System (CCRS) is a national information system developed and managed by the Canadian Institute for Health Information (http://www.cihi.ca). Originally developed as the Ontario Chronic Care Patient System (OCCPS) to support the implementation of the RAI 2.0 in CCC hospitals/units, the CCRS now serves as a pan-Canadian data repository for eight participating provinces/territories implementing this instrument. It is a vehicle for national statistical reporting on the status of continuing care facilities (see, for example, CIHI report [20]), providing a national perspective on facility-based services for frail elderly and persons with disabilities.

Besides making the reporting system available to other provinces, the launch of the CCRS in 2003 included minor updates to the RAI 2.0 form and coding instructions. Also, forms were added to track demographic changes and to obtain a profile of participating facilities. Currently, all CCC hospital patients and LTC residents are assessed within 14 days of admission. In addition, quarterly re-assessments are done using a shortened form and annual re-assessments are done using the full MDS form. A discharge tracking form is used to record the date and disposition of all discharges, but a full assessment is not done at discharge. Data are sent electronically to the CCRS and must adhere to reporting standards established by CIHI in consultation with the provinces and interRAI. This includes passing new logical checks for data submissions. Seven of the eight provinces/territories that are implementing the RAI 2.0 are now submitting data to CIHI. Data are available for Ontario CCC hospitals/units beginning in 1996 and for LTC in that province as of 2005. More detailed descriptions of the implementation of the RAI 2.0 in Canada, beginning with CCC hospital settings in Ontario, are provided elsewhere [4,21-28].

With the growing importance of the CCRS for decision-making in continuing care, questions about the system’s data quality have naturally arisen. The integrity of the data system is a precondition of its acceptance as a basis for these decisions. Therefore, there has been growing interest in current data quality of the CCRS and how it has changed over time. Also, there is interest in identifying methods that might be used to monitor, identify, and respond to changes in data quality in the future.

### The changing role of facilities in the continuum of care

A variety of factors related to data quality could have introduced *measurement error* into the data over time, including changes in the population composition and the roles of CCC and LTC in the continuum of care. Therefore, any assessment of data quality must consider strategies for disaggregating indications of error from measures of actual changes in practice patterns or population characteristics in the sector of interest. Hirdes et al. [23] discuss the impact of the Health Services Restructuring Commission (HSRC) on the role of CCC and LTC. The HSRC provided a clear policy directive that CCC should reduce its emphasis on long-stay medically stable patients in favour of an increased emphasis on post-acute care for persons needing physical rehabilitation or complex medical care. The RUG-III algorithm was used to specify cutoffs for the two care settings. The bulk of the RUG-III categories referencing Impaired Cognition, Behaviour Disturbance, and Reduced Physical Functions were designated for LTC. The remaining categories (Rehabilitation, Extensive Services, Special Care and Clinically Complex) were designated for CCC. One of the most profound changes that has occurred has been a substantial reduction of length of stay in CCC, dropping from an average of 224 days in 1996 to less than 90 days by 2010 [4,29]. In fact, CCC can be best thought of as containing two fairly distinct populations: a) short-stay post-acute patients who tend to be discharged within 90 days; and b) long stay medically complex patients who comprise a larger portion of all patient days while nevertheless representing only about 20 percent of admissions. LTC, on the other hand, tend to have a more stable population with longer stays but substantial impairments in cognition or functional status.

### Types of data quality issues

As noted previously, psychometric testing to show reliability and validity of an assessment instrument is a necessary condition for its use as a basis for decision-making. In response to early concerns about the suitability of RAI data for research use and other health care decision-making (e.g., [30-32], the RAI 2.0 and related interRAI instruments have been subject to extensive, on-going testing to establish reliability [33-36] and validity [37-44]. A more detailed review of this evidence is available elsewhere [45]. However, the performance of an assessment instrument in a research context may not be matched by a similar level of reliability and validity when used as part of normal clinical practice [46]. For example, Crooks and colleagues [47] identified low agreement between urinary continence ratings done as part of normal clinical practice and subsequent independent tests of wetness done by research staff. These were explained, at least in part, by poor assessment practices that would be problematic with the implementation of any instrument, no matter how it performed in research trials. Therefore, it is necessary to identify potential threats to data quality in normal use and to develop methods to appraise the extent to which such problems are evident in the data obtained as part of regular clinical practice.

#### Random error

All assessment instruments will have some random error in which the “test score” obtained by its items will have some level of disagreement with the “true score” for the person who has been assessed. For example, when measuring behaviour based on events that can occur at different times over the course of the day (e.g., angry outbursts), there will be chance variations in whether the events were witnessed by staff or other informants. Characteristics that are more stable over time (e.g., activities of daily living) will have lower levels of random error than those that are relatively more volatile or changeable over time (e.g., fever, delirium, depression, behavior disturbance).

Random error is a source of concern with data quality, because it tends to attenuate the associations between variables of interest. Thus, this error makes it more difficult to detect true differences between populations or to identify relationships between variables. For example, higher levels of random error will weaken evidence of a relationship between a best practice intervention and a desired outcome and it may also create false regional differences in resource intensity or quality of care.

The conventional tests to evaluate random error include measures of reliability such as inter-rater reliability (tests of agreement between two independent raters) and tests of internal consistency (tests of the item correlations within parallel form scales).

#### Systematic error

A more troublesome form of error introduces a bias for either intentional or unintentional reasons. While random error may reduce the ability to detect associations by increasing “noise,” systematic error may alter associations such that they are not valid reflections of the true relationship between the variables being studied. For example, if an assessor believes that the specific responses on a given item may have negative organizational consequences, the assessor may have an incentive to record a response that would change the likelihood of those consequences occurring.

A specific example that is a worrisome problem for any health information system is the gaming of the case-mix algorithms that inform funding decisions. In this case, assessors may bias their coding practices in favour of more severe ratings on items used in the case-mix system with the aim of getting higher ratings of resource intensity. Gaming has many negative consequences, including the undermining of credibility of the entire information system to the point that participating organizations feel they cannot trust the data from their peer organizations. Unchecked gaming can also lead to the unfair allocation of limited resources if higher severity ratings are the product of biased reporting rather than the residents’ true need.

A distinction should be made between efforts to game a case-mix system and changes in practice patterns resulting from incentives in a case-mix system (e.g., the actual provision of more rehabilitation care, because it is paid at its cost or more). While the latter issue might be of concern for other reasons, it is not the same as the deliberate misrepresentation of resident characteristics in the pursuit of economic or other advantages.

Ascertainment bias is a different type of systematic error that is the result of differences in staff efforts or expertise in detecting difficult to measure resident characteristics. For example, pain, depression, and delirium are subtle, rather complex problems to detect, particularly in populations with substantial impairments in cognition or communication. Staff members who are skilled, sensitive or diligent in assessing these characteristics will detect higher rates of the problem than others who are less adept. Conversely, poor assessment practices may result in a systematic failure to detect a problem. Ascertainment bias can also be the product of preconceived notions that staff may have about the presence or absence of traits in certain types of patients (e.g., activity preferences and cognitive impairment). This type of bias is of concern because it may lead to a failure to detect problems in subpopulations that are difficult to assess (e.g., persons with dementia), and organizational variations may lead to false conclusions about differences in quality of care.

A third type of systematic error involves social desirability bias. In this case, reporting on assessment systems may be biased to avoid negative impressions of the resident, staff member or organization. For example, there may be a tendency to understate true quality problems if it is seen to be a reflection of the individual personally or on the managers of the organization.

#### Selection bias

Selection bias is typically considered to be a problem in sample data where certain types of individuals may be systematically included or excluded from a data set. However, it will also be a concern in organizational comparisons of quality when facilities tend to admit patients with different histories of potential quality problems. These past differences may also translate into differential likelihoods of the quality problem in the future in the absence of differences in clinical practice. A different type of selection bias may occur when there is discretion exercised about who does or does not receive an assessment (e.g., organizational differences in the rate of late RAI 2.0 assessments).

#### Autopopulation

Autopopulation refers to the practice of using data from another source to complete the fields of a current assessment, automatically and with no further scrutiny. Autopopulation would include the use of items from one instrument to fill out fields of a different instrument, or it may involve the use of old records from an assessment to complete the same fields in a new assessment. Hirdes et al. [48] explored the options for autopopulation of MDS items over time and concluded there were only a few fields where one might confidently carry forward prior values.

Autopopulation can cause many, serious data quality problems for a health information system. For example, it can make the data set unresponsive to true change if values are carried forward without clinician confirmation that no change has occurred. This, in turn, can reduce the evidence of the effectiveness of interventions or therapies on outcomes of interest. Moreover, it would mask good and poor performance on outcome-based quality indicators, because it would suppress evidence of changes in those measures.

#### Data completeness

Item non-response can result in data sets that are so incomplete as to render them worthless. Missing values (and items with an “Unknown” response) can make an observation unusable, thereby reducing the sample size in any analysis or report. While some solutions for dealing with missing values have been proposed (e.g., imputation based on other scores, substitution of median or mean scores) these approaches often are unacceptable (e.g., if funding or quality performance decisions are based on those data). Increases in the magnitude of item non-response will make a data set less representative over time, which is a serious problem for smaller organizations where the loss of data on a few cases could substantially alter the estimates of rates of a quality problem. Despite the serious threat posed by missing data, there are several fairly easy solutions to the problem. Examples include educational strategies to sensitize staff to the problem, instrument design to reduce the risk of ambiguity leading to non-response, and the use of electronic data checks to ensure that all fields are complete.

#### Logical errors

Logical errors include several types of coding inconsistencies that can result from poor assessment practices, poor instrument design, assessor fatigue, systematic biases, or random error. First, they include discrepancies between variables whose values are contingent on each other (e.g., items in a list are checked and “none of the above” is checked). Second, there may be out of range values (e.g., heights or weights scored in units not consistent with national standards or coding responses not associated with any meaning). Third, there may be improbable or impossible combinations of items (e.g., comatose but rated as engaging in group activities; elimination of chronic diseases for which there is no known cure; readmission dates that fall before admission dates). Among the problems caused by logical errors is the distortion of observations to be the equivalent of missing values. For example, if two responses cannot logically both be true, it is unclear which of the two *is* true. Hence, the problems with data completeness may also apply to logical errors.

### Study objectives

The present study aims to examine the quality of CCRS data based on the Ontario CCC and LTC data submitted to CIHI between 1996–2011 and 2005–2011, respectively. It considers changes in population characteristics and practice patterns, and examines the rate of multiple potential quality problems. This study also aims to evaluate analytic strategies that may be used to evaluate data quality in other similar data sets (e.g., the RAI-Home Care and RAI-Mental Health, both of which have been implemented in other sectors or jurisdictions). The focus is on Ontario even though CCRS is a national reporting system because that province has the longest and most complete implementation of the RAI 2.0 at the time of writing.

## Methods

### Data source

The 2010–2011 CCRS data, received from CIHI with encrypted facility and resident identifiers, was used for analysis. This dataset contained assessments done from July 1, 1996 to March 31, 2011 in both Ontario CCC hospitals/units (n = 466,767) and LTC (n = 900,885) for a total of 1,367,652 assessments. Because of their unique nature, individuals assessed as comatose were excluded. The dataset was then sorted by assessment date, assigned to a quarter, and analyses were stratified by sector. All assessments were used, including admission, quarterly and annual reassessments. There were 181,548 individuals assessed in CCC settings in that time period compared with 135,245 in LTC after implementation began in 2005 until 2011 when it was complete. With longer stay in the latter, the average number of assessments per individual was 2.6 in CCC and 6.7 in LTC.

### Analysis

The primary analytic approach was to examine the quarterly time series trends for various indicators. Stratified analyses were done where differences were evident between CCC hospitals/units and LTC. Linear regression models were fitted to the various time series to estimate the magnitude of change in the selected indicators over time. Various indicators of population change in clinical characteristics, service utilization, and resource intensity were considered. The data were examined for trends in measures of convergent validity using measures of association (e.g., Pearson’s r, Cramer’s V) for variables expected to be related to each other where the relationship is unlikely to change dramatically over time (e.g., cognitive impairment and ADL status). This is an extension of the approach used by Phillips and Morris [45] in their analyses of US MDS 2.0 data.

To examine reliability in the CCRS, Cronbach’s alpha was used as a measure of internal consistency for three parallel form scales embedded in the RAI 2.0: the Activities of Daily Living Scale - Long Form [49]; Depression Rating Scale [50] and the Aggressive Behavior Scale [51]. This subset of available interRAI scales was selected because they are likely to have different levels of reliability based on previous research (e.g., the ADL scale tends to have high Cronbach’s alpha scores, whereas the DRS tends to be at the lower end of the acceptable range of reliability). These differences are useful for calibrating the degree of decline (or improvement) in internal consistency for scales known to have different baseline levels of reliability. Although the Cognitive Performance Scale [52] was used to examine associations with other variables, its reliability could not be evaluated with Cronbach’s alpha, because it is not a parallel form scale. Instead it is a decision-tree algorithm that was derived using diverse correlates of cognition in predictive models.

Some of CIHI’s rules for logical inconsistencies in coding practices were considered for both clinical and service utilization indicators. In addition, new longitudinal indicators of logical errors, based on the failure to code diagnoses at follow-up that were present at baseline and were unlikely to have been cured (e.g., multiple sclerosis), were developed by an expert panel of clinicians, interRAI researchers and CIHI staff. Three measures of potential autopopulation were constructed by examining the absence of change in sets of indicators over time. ADL function and mood indicators were chosen to represent two clinical domain areas with different patterns of stability and change over time. Finally, the time between the assessment reference date and the date the assessment was signed off as complete, was considered as a measure of efficiency in completing the assessment and of the appropriateness of assessment practices.

#### Ethics clearance

The study was reviewed and received ethics clearance through the Office of Research Ethics at the University of Waterloo (ORE #13848).

## Results

Table 1 shows a number of key descriptive characteristics for the population studied based on all available assessments in the study period. They include percentage estimates for: admission assessments, Cognitive Performance Scale (CPS) scores of three or more (indicating moderate or worse cognitive impairment), Activities of Daily Living (RUG ADL) scores of 11 or more (indicating moderate or worse functional impairment based on the scale used in the RUG-III case-mix system), and Aggressive Behaviour Scale (ABS) scores of five or more (indicating a high-level of behaviour disturbance), all for each quarter from the third quarter in 1996 to the first quarter in 2011, inclusive. Each of these indicators was stratified by type of facility, as there were notable differences evident. There was a consistently higher percentage of admission assessments in the CCC hospitals/units than in LTC over the entire 59-quarter study period with observations for both sectors. The percentage of admission assessments increased over time in CCC reflecting a policy shift toward a greater emphasis on post-acute care during the 15 year study period for that sector. There was only modest change in the proportion of admission assessments in LTC, which serve a longer staying, more stable population. The higher proportion of admission assessments in Quarter 2 and 3 of 2006 reflect an administrative artifact of LTC beginning implementation of the RAI 2.0.

**Table 1:** Percentage of persons with selected characteristics over time, by facility type (CCC and LTC), Ontario *

**Quarter**	**Admission assessments**		**Cognitive performance scale 3+**		**RUG ADL scale 11+**		**Aggressive behaviour scale 5+**		**Depression rating scale 3+**		**Number of assessments**	
	**CCC**	**LTC**	**CCC**	**LTC**	**CCC**	**LTC**	**CCC**	**LTC**	**CCC**	**LTC**	**CCC**	**LTC**
**1996_3**	32.9%		64.2%		75.0%		11.5%		24.6%		9,164	NA
**1996_4**	28.7%		64.0%		75.1%		10.6%		23.6%		8,604	NA
**1997_1**	30.1%		63.4%		74.5%		10.7%		22.8%		8,372	NA
**1997_2**	33.2%		62.7%		73.4%		10.6%		22.7%		7,776	NA
**1997_3**	30.9%		63.4%		74.5%		10.6%		22.5%		8,199	NA
**1997_4**	32.6%		62.7%		74.1%		10.4%		22.8%		8,067	NA
**1998_1**	34.4%		61.5%		73.5%		9.8%		23.1%		8,103	NA
**1998_2**	34.8%		61.5%		73.3%		10.2%		22.5%		7,913	NA
**1998_3**	33.8%		61.6%		73.3%		9.5%		22.6%		7,855	NA
**1998_4**	36.3%		61.1%		73.1%		9.4%		22.4%		8,024	NA
**1999_1**	37.8%		60.4%		72.1%		9.3%		22.1%		7,977	NA
**1999_2**	36.0%		60.0%		72.0%		9.4%		22.5%		7,887	NA
**1999_3**	35.4%		60.3%		72.7%		9.0%		22.6%		7,798	NA
**1999_4**	37.0%		60.1%		73.0%		8.9%		23.3%		7,883	NA
**2000_1**	37.9%		60.8%		73.2%		9.2%		24.2%		7,899	NA
**2000_2**	39.0%		60.1%		72.9%		9.0%		24.7%		7,840	NA
**2000_3**	37.9%		59.4%		72.4%		9.1%		24.3%		7,795	NA
**2000_4**	41.1%		58.7%		72.2%		8.5%		24.5%		7,607	NA
**2001_1**	42.8%		58.8%		72.5%		8.3%		24.0%		7,908	NA
**2001_2**	42.7%		59.1%		73.4%		8.4%		24.6%		7,903	NA
**2001_3**	41.9%		59.4%		74.0%		9.0%		26.5%		7,850	NA
**2001_4**	43.0%		58.3%		73.6%		9.3%		26.7%		8,020	NA
**2002_1**	42.7%		59.0%		73.5%		9.8%		27.0%		7,909	NA
**2002_2**	43.5%		58.4%		73.5%		8.7%		26.4%		7,640	NA
**2002_3**	44.5%		57.8%		73.6%		8.3%		26.8%		7,718	NA
**2002_4**	45.3%		57.6%		72.1%		7.8%		26.8%		7,732	NA
**2003_1**	45.2%		58.4%		73.0%		7.7%		27.4%		7,572	NA
**2003_2**	49.3%		57.8%		71.5%		7.8%		26.2%		7,431	NA
**2003_3**	49.8%		55.9%		71.1%		7.5%		26.5%		7,535	NA
**2003_4**	47.4%		54.4%		71.0%		6.9%		26.3%		7,748	NA
**2004_1**	48.3%		54.3%		70.7%		6.9%		25.7%		7,724	NA
**2004_2**	48.0%		54.2%		70.6%		6.7%		26.0%		7,662	NA
**2004_3**	49.0%		53.2%		69.9%		6.9%		24.5%		7,745	NA
**2004_4**	49.7%		53.7%		69.5%		6.6%		24.3%		7,718	NA
**2005_1**	51.4%		52.2%		69.6%		6.5%		24.6%		7,792	NA
**2005_2**	50.9%		53.3%		68.9%		6.5%		25.2%		7,612	NA
**2005_3**	50.8%	6.8%	52.9%	64.2%	70.6%	62.4%	6.8%	19.3%	26.1%	31.6%	7,438	1,373
**2005_4**	51.9%	8.4%	52.4%	62.7%	70.0%	57.3%	6.6%	18.4%	24.2%	33.6%	7,519	2,699
**2006_1**	52.1%	9.3%	52.1%	61.9%	69.6%	56.6%	6.6%	16.5%	25.0%	34.1%	7,476	2,906
**2006_2**	48.0%	21.3%	51.3%	60.9%	70.1%	57.4%	7.0%	15.2%	26.5%	35.0%	7,435	5,825
**2006_3**	45.8%	22.4%	52.6%	61.2%	71.3%	59.0%	7.1%	14.4%	26.6%	35.2%	7,164	13,428
**2006_4**	46.6%	8.5%	52.3%	62.0%	70.4%	60.2%	7.5%	14.1%	26.5%	36.0%	7,149	13,589
**2007_1**	49.1%	9.7%	51.9%	60.8%	71.3%	60.0%	7.2%	13.9%	27.2%	35.1%	7,241	13,538
**2007_2**	49.6%	9.0%	51.3%	60.0%	71.6%	58.9%	7.4%	13.3%	27.9%	34.1%	7,190	14,337
**2007_3**	47.8%	8.7%	51.0%	59.1%	70.4%	58.9%	7.7%	12.2%	27.5%	32.5%	7,077	18,231
**2007_4**	49.3%	8.9%	49.7%	58.7%	71.2%	57.7%	7.4%	12.1%	26.4%	32.4%	7,231	21,999
**2008_1**	48.2%	9.4%	50.9%	58.1%	70.8%	56.8%	7.7%	11.6%	26.8%	31.6%	7,137	22,307
**2008_2**	50.0%	8.9%	50.5%	58.1%	71.7%	57.1%	7.0%	11.8%	24.9%	30.8%	7,097	28,323
**2008_3**	47.2%	7.8%	50.6%	58.5%	72.1%	57.4%	7.1%	11.3%	25.5%	30.1%	6,898	30,750
**2008_4**	49.5%	8.5%	49.2%	58.0%	72.2%	57.4%	6.6%	11.0%	24.7%	29.0%	7,038	31,373
**2009_1**	50.0%	8.7%	50.0%	57.3%	71.9%	57.1%	6.7%	10.5%	24.9%	27.7%	6,928	31,305
**2009_2**	53.4%	8.5%	49.5%	57.9%	70.4%	57.9%	6.7%	11.1%	24.3%	29.6%	7,124	40,454
**2009_3**	51.4%	7.4%	50.4%	58.3%	71.3%	58.5%	6.9%	11.8%	24.2%	32.7%	6,947	61,281
**2009_4**	51.9%	8.1%	49.5%	58.0%	71.0%	59.2%	6.4%	12.0%	23.1%	33.4%	7,192	74,405
**2010_1**	50.7%	8.8%	48.3%	57.7%	70.2%	59.8%	6.3%	12.0%	22.8%	32.9%	7,026	77,427
**2010_2**	51.9%	8.6%	47.0%	57.5%	70.3%	60.0%	5.8%	11.5%	22.7%	32.2%	6,954	77,554
**2010_3**	52.2%	8.1%	47.4%	57.8%	70.2%	60.6%	6.1%	11.3%	23.2%	31.4%	6,979	78,103
**2010_4**	54.9%	9.0%	45.4%	58.0%	68.9%	60.9%	5.7%	11.2%	22.6%	31.5%	7,032	78,497
**2011_1**	56.6%	9.3%	47.0%	58.3%	70.3%	61.6%	5.6%	11.2%	23.1%	31.8%	6,943	78,255
**Intercept**	32.8%	12.0%	64.4%	62.3%	74.0%	58.0%	10.3%	16.4%	23.9%	34.1%	–	–
**Slope**	0.39%	-0.19%	-0.30%	-0.25%	-0.07%	0.07%	-0.08%	-0.29%	0.03%	-0.15%	–	–

*Note: in this and subsequent tables, adequate numbers of LTC assessments for analysis were only available starting in the third quarter of 2005.

With respect to clinical attributes, the prevalence of moderate or worse cognitive impairment was notably higher in LTC compared with CCC. There was an absolute reduction by 17% of patients with that level of cognitive impairment in CCC, whereas that subgroup remained relatively stable at about 60% of the LTC population. The percentage with moderate or worse ADL impairment remained stable in both settings over time and was consistently higher in the CCC hospitals/units. The differences in the ABS scores were modest between the two facility types over time, with a somewhat higher proportion with severe behavior disturbance in LTC. The percentage of LTC residents with high ABS scores was greater among early adopter homes, but was relatively stable at about 12% by the final quarter of 2007. The percentage with a DRS score of three or more was stable over time in both settings, but tended to be about 10% higher in LTC compared with CCC.

Table 2 reports on measures of service utilization and resource intensity by facility type, including mean rehabilitation therapy minutes (i.e., the total minutes of speech, occupational and physical therapy received in the last 7 days), percentage of patients receiving two or more nursing rehabilitation interventions (includes passive and active range of motion exercises; splint or brace assistance; training/skill practice in bed mobility, transfer, walking, dressing or grooming, eating or swallowing, amputation or prosthesis care, scheduled toileting/bladder retraining, and communication), and mean RUG-III Case-Mix Index (CMI). For both settings, there were a notable increases in reported therapy minutes per week and the proportion reported to receive two or more nursing rehabilitation requirements. In CCC, the mean rehabilitation minutes rose from about 66 minutes when the RAI 2.0 was mandated to about 143 minutes in the first quarter of 2011. In LTC they rose from about 16 minutes in 2005 to 42 minutes in 2011. Similarly, the percentage of CCC patients receiving nursing rehabilitation increased from about 27% at the start of the study period to about 64% and from about 10% in 2006 to 26% in LTC. The mean CMIs were notably higher in all years for CCC compared with LTC, reflecting differences in the populations served and the intensity of interventions received.

**Table 2:** Trends in clinical interventions and resource intensity over time, by facility type (CCC and LTC), Ontario

	**Mean therapy minutes**		**2+ Nursing rehabilitation procedures 6+ days/week**		Mean 2004 case mix index*		**Mean current case mix index**		**Receipt of any occupational therapy**		**Receipt of any Physical Therapy**		**3+ Days rehabilitation & 45+ minutes/week**		**5+ Days rehabilitation & 150+ minutes/week**		**Special rehabilitation RUG-III level**		**Number of assessments**	
**Quarter**	**CCC**	**LTC**	**CCC**	**LTC**	**CCC**	**LTC**	**CCC**	**LTC**	**CCC**	**LTC**	**CCC PT**	**LTC PT**	**CCC**	**LTC**	**CCC**	**LTC**	**CCC**	**LTC**	**CCC**	**LTC**
**1996_3**	66.0		26.8%		0.89		0.99		23.9%		37.4%		29.7%		12.8%		19.2%		9,164	NA
**1996_4**	64.3		28.2%		0.89		1.00		23.7%		38.1%		29.8%		12.5%		19.3%		8,604	NA
**1997_1**	65.6		28.5%		0.89		1.00		23.1%		38.3%		30.2%		13.0%		19.2%		8,372	NA
**1997_2**	75.8		29.5%		0.90		1.00		25.7%		39.6%		32.5%		15.0%		21.9%		7,776	NA
**1997_3**	72.2		29.6%		0.90		1.00		26.4%		41.2%		33.5%		15.0%		21.8%		8,199	NA
**1997_4**	75.2		32.8%		0.91		1.01		29.2%		41.6%		34.8%		15.3%		23.2%		8,067	NA
**1998_1**	80.7		33.5%		0.92		1.02		31.2%		44.3%		36.7%		16.6%		24.5%		8,103	NA
**1998_2**	81.8		35.1%		0.92		1.02		31.1%		44.9%		36.9%		16.7%		25.4%		7,913	NA
**1998_3**	80.8		34.5%		0.92		1.02		29.1%		44.7%		36.4%		16.4%		25.0%		7,855	NA
**1998_4**	80.2		33.9%		0.92		1.03		29.0%		45.5%		36.8%		16.7%		24.5%		8,024	NA
**1999_1**	89.8		35.6%		0.92		1.03		30.3%		46.3%		37.1%		16.5%		24.8%		7,977	NA
**1999_2**	80.2		35.2%		0.92		1.02		30.3%		46.6%		36.2%		16.0%		24.7%		7,887	NA
**1999_3**	80.0		35.3%		0.93		1.03		31.2%		47.3%		37.4%		17.0%		25.6%		7,798	NA
**1999_4**	82.2		37.3%		0.93		1.04		32.1%		47.9%		38.3%		16.7%		26.2%		7,883	NA
**2000_1**	86.2		39.2%		0.94		1.05		35.3%		48.4%		39.9%		18.4%		27.9%		7,899	NA
**2000_2**	84.0		39.6%		0.95		1.06		34.5%		50.0%		40.6%		18.4%		28.6%		7,840	NA
**2000_3**	87.2		40.3%		0.95		1.05		33.2%		47.5%		38.9%		17.5%		27.8%		7,795	NA
**2000_4**	87.6		41.9%		0.95		1.05		34.5%		49.2%		40.0%		17.8%		28.3%		7,607	NA
**2001_1**	85.2		42.3%		0.96		1.07		35.9%		49.2%		40.5%		18.1%		29.1%		7,908	NA
**2001_2**	88.0		49.3%		0.97		1.08		36.6%		48.8%		40.8%		18.0%		30.8%		7,903	NA
**2001_3**	85.4		53.8%		0.97		1.08		35.4%		49.3%		40.4%		17.7%		31.4%		7,850	NA
**2001_4**	85.7		57.8%		0.99		1.10		35.9%		51.7%		42.2%		18.5%		33.7%		8,020	NA
**2002_1**	88.6		61.4%		0.99		1.10		36.6%		53.4%		44.1%		19.3%		36.1%		7,909	NA
**2002_2**	98.8		64.4%		1.00		1.11		38.5%		53.8%		45.1%		20.3%		37.6%		7,640	NA
**2002_3**	96.4		63.9%		1.00		1.11		36.7%		53.2%		44.1%		20.7%		37.2%		7,718	NA
**2002_4**	96.9		64.7%		1.00		1.11		39.6%		55.2%		45.9%		20.6%		38.0%		7,732	NA
**2003_1**	97.3		65.7%		1.01		1.13		40.0%		55.5%		45.6%		21.0%		39.1%		7,572	NA
**2003_2**	100.2		65.9%		1.01		1.04		40.6%		55.7%		46.4%		21.6%		39.9%		7,431	NA
**2003_3**	95.8		65.2%		1.00		1.03		39.7%		56.2%		46.1%		20.5%		39.1%		7,535	NA
**2003_4**	98.9		64.9%		1.01		1.04		42.5%		56.8%		47.8%		22.1%		40.9%		7,748	NA
**2004_1**	106.4		64.9%		1.01		1.05		43.4%		58.9%		50.3%		24.1%		43.4%		7,724	NA
**2004_2**	108.6		65.2%		1.02		1.03		44.8%		60.6%		51.3%		24.6%		44.2%		7,662	NA
**2004_3**	110.3		66.0%		1.02		1.02		45.3%		59.7%		51.0%		25.3%		44.2%		7,745	NA
**2004_4**	110.7		64.1%		1.02		1.03		46.3%		60.9%		51.2%		25.3%		44.0%		7,718	NA
**2005_1**	112.1		65.7%		1.02		1.03		46.3%		60.7%		51.6%		25.9%		44.9%		7,792	NA
**2005_2**	117.8		66.7%		1.03		1.03		48.2%		62.7%		53.3%		27.5%		46.3%		7,612	NA
**2005_3**	118.8	14.8	66.9%	23.1%	1.03	0.70	1.04	0.70	51.8%	2.3%	63.6%	32.1%	54.7%	13.0%	28.4%	0.2%	47.9%	2.4%	7,438	1,373
**2005_4**	116.1	10.3	66.2%	14.1%	1.03	0.68	1.03	0.68	52.4%	1.4%	62.9%	23.3%	54.4%	9.9%	27.4%	0.3%	47.7%	1.8%	7,519	2,699
**2006_1**	119.4	10.2	66.3%	12.9%	1.04	0.68	1.04	0.68	53.1%	1.5%	64.5%	21.9%	55.5%	8.9%	28.9%	0.3%	48.8%	1.5%	7,476	2,906
**2006_2**	121.6	19.2	68.5%	9.4%	1.04	0.68	1.03	0.67	53.5%	3.2%	64.6%	35.5%	55.7%	16.5%	28.5%	0.4%	49.5%	1.6%	7,435	5,825
**2006_3**	117.2	22.9	67.9%	8.3%	1.04	0.69	1.03	0.67	50.8%	2.6%	63.7%	41.7%	53.7%	19.3%	27.8%	0.5%	47.9%	2.1%	7,164	13,428
**2006_4**	119.0	22.7	68.6%	9.3%	1.04	0.69	1.03	0.68	52.9%	3.2%	63.9%	43.7%	54.7%	17.4%	28.2%	0.6%	48.6%	2.5%	7,149	13,589
**2007_1**	114.7	24.7	67.9%	11.5%	1.04	0.69	1.03	0.68	51.2%	2.9%	63.3%	45.6%	53.8%	17.0%	27.0%	0.4%	48.0%	2.8%	7,241	13,538
**2007_2**	120.1	24.6	68.4%	14.6%	1.05	0.70	1.02	0.67	52.7%	3.1%	65.0%	47.8%	55.5%	18.8%	28.3%	0.6%	48.9%	4.0%	7,190	14,337
**2007_3**	118.6	25.2	69.9%	15.1%	1.04	0.69	1.01	0.67	52.6%	2.5%	64.0%	49.1%	55.1%	19.5%	27.9%	0.8%	49.0%	4.9%	7,077	18,231
**2007_4**	121.4	25.2	69.1%	15.5%	1.05	0.69	1.01	0.66	53.6%	2.0%	65.2%	49.6%	55.9%	19.9%	29.4%	0.8%	49.4%	4.9%	7,231	21,999
**2008_1**	119.5	25.0	69.0%	15.0%	1.05	0.69	1.02	0.66	54.2%	2.0%	65.0%	49.2%	56.6%	20.9%	29.5%	0.9%	49.8%	5.0%	7,137	22,307
**2008_2**	122.4	26.0	68.6%	13.9%	1.05	0.69	1.01	0.65	53.5%	2.4%	66.2%	50.5%	57.4%	22.6%	30.8%	0.9%	50.5%	5.2%	7,097	28,323
**2008_3**	117.4	27.3	69.2%	13.5%	1.04	0.69	1.00	0.65	53.8%	3.0%	65.9%	54.3%	56.9%	24.5%	28.7%	0.8%	49.9%	5.4%	6,898	30,750
**2008_4**	124.6	27.6	67.2%	13.9%	1.05	0.69	1.01	0.65	56.2%	3.3%	68.2%	55.2%	59.1%	25.6%	31.5%	1.1%	51.5%	6.5%	7,038	31,373
**2009_1**	127.2	28.5	66.9%	14.6%	1.06	0.70	1.01	0.66	56.6%	3.3%	68.2%	57.4%	59.5%	27.8%	32.5%	1.2%	51.6%	7.2%	6,928	31,305
**2009_2**	128.5	31.3	68.1%	13.8%	1.05	0.70	1.00	0.66	57.1%	3.0%	68.6%	61.6%	60.0%	31.1%	32.0%	1.1%	51.9%	7.4%	7,124	40,454
**2009_3**	122.2	33.3	69.6%	13.3%	1.05	0.70	1.00	0.66	55.1%	2.8%	67.3%	64.9%	58.6%	35.8%	30.9%	1.2%	51.3%	8.0%	6,947	61,281
**2009_4**	123.2	35.6	69.0%	16.4%	1.04	0.72	1.00	0.67	54.4%	2.8%	66.9%	68.7%	58.6%	41.8%	30.0%	1.3%	50.6%	11.2%	7,192	74,405
**2010_1**	127.9	38.1	68.8%	22.2%	1.05	0.73	1.00	0.69	55.9%	3.5%	69.1%	73.1%	60.6%	49.4%	31.5%	1.2%	52.4%	15.7%	7,026	77,427
**2010_2**	133.4	40.6	67.6%	25.6%	1.05	0.75	1.00	0.70	57.5%	3.7%	69.6%	77.5%	61.8%	56.7%	32.4%	1.3%	53.5%	19.8%	6,954	77,554
**2010_3**	130.7	41.0	68.3%	27.7%	1.04	0.75	0.99	0.71	56.6%	3.4%	69.9%	79.2%	61.3%	59.1%	31.5%	1.2%	52.7%	21.9%	6,979	78,103
**2010_4**	137.6	41.8	67.4%	28.2%	1.05	0.76	1.00	0.71	57.1%	3.5%	71.1%	80.1%	61.9%	61.9%	33.9%	1.2%	54.1%	22.6%	7,032	78,497
**2011_1**	142.0	42.4	64.3%	26.7%	1.06	0.76	1.01	0.71	58.5%	3.3%	71.5%	81.1%	63.2%	63.2%	34.8%	1.3%	54.1%	21.4%	6,943	78,255
**Intercept**	67.5	11.9	32.0%	9.5%	0.90	0.67	1.05	0.66	23.9%	2.1%	39.4%	24.2%	30.8%	1.3%	12.3%	0.2%	19.4%	-3.1%	–	–
**Slope**	1.17	1.32	0.79%	0.58%	0.00	0.00	0.00	0.00	0.63%	0.06%	0.57%	2.49%	0.56%	2.36%	0.37%	0.05%	0.65%	0.93%	–	–

*For comparability with previous analyses, the CMI was recalculated using 2004 values.

Table 3 reports the distributions of facility-level percentages of persons qualifying for the RUG-III Special Rehabilitation level in the 2008 to 2011 time period. Although there are changes in both sectors, the changes in the percentage of LTC residents at this RUG-III following its inclusion in the payment system for that sector are striking. Surprisingly, the RUG-III distributions for about 10 percent of LTC suggested levels of Special Rehabilitation that were evident in rehabilitation hospitals, funded at substantially higher rates.

**Table 3:** Percentage distributions of residents that qualify at the Special Rehabilitation RUG-III level, by facility type (CCC and LTC), Ontario, 2008 - 2011

	**Complex continuing care**				**Long term care**			
**Year**	**2008**	**2009**	**2010**	**2011**	**2008**	**2009**	**2010**	**2011**
**Number of facilities**	122	119	115	106	221	626	631	632
**10**^**th**^**percentile**	0.0%	0.0%	0.0%	0.0%	0.0%	0.0%	0.0%	0.0%
**25**^**th**^**percentile**	13.3%	9.0%	17.9%	12.7%	0.0%	0.0%	1.2%	1.6%
**median**	39.4%	43.6%	50.0%	50.0%	0.6%	0.9%	8.3%	13.7%
**75**^**th**^**percentile**	61.2%	65.1%	62.8%	66.7%	4.6%	6.9%	30.2%	34.5%
**90**^**th**^**percentile**	75.2%	76.7%	80.8%	83.7%	15.9%	26.2%	58.9%	55.6%

Whereas the previous tables can be presumed, at least in part, to reflect changes in the CCC and LTC populations and the services they received over time, Table 4 considers the patterns of associations (an indicator of convergent validity) intended to yield insight into data quality of clinical elements in the RAI 2.0. The relationships of four variables with cognitive impairment are examined to identify the magnitude and direction of their associations; and to examine the stability of these associations over time. Both ADL impairment and aggressive behaviour are positively correlated with the CPS, but the relationship is strongest for ADL. Similarly, using the Cramer’s V statistic for associations in cross-tabulations, bowel incontinence is positively related to cognitive impairment. On the other hand, there is a negative correlation of the interRAI Pain Scale with the CPS. This is not surprising given the wealth of literature that point to this negative relationship, which is often explained in part by under-detection of pain in cognitively impaired patients Proctor and Hirdes [53]). To the extent that this latter association is a function of ascertainment bias, one might expect to see some change in this association as practice patterns improve. While there is almost no change in the associations of CPS with ADL, bowel continence, and aggressive behaviour over time, there was a slight weakening of the correlation of CPS and pain in the study period. There were only modest between-sector differences, suggesting that the associations of these clinical variables were relatively stable between LTC and CCC.

**Table 4:** Trends in indicators of convergent validity for selected variables over time, by facility type (CCC and LTC), Ontario

	**Bowel**		**ADL & CPS**		**Pain & CPS**		**ABS & CPS**		**Number of assessments**	
	Continence & CPS 3+ *		Correlation**		Correlation**		Correlation**			
**Quarter**	**CCC**	**LTC**	**CCC**	**LTC**	**CCC**	**LTC**	**CCC**	**LTC**	**CCC**	**LTC**
**1996_3**	0.42		0.59		-0.25		0.28		9,164	NA
**1996_4**	0.41		0.58		-0.23		0.30		8,604	NA
**1997_1**	0.43		0.59		-0.22		0.30		8,372	NA
**1997_2**	0.43		0.58		-0.20		0.31		7,776	NA
**1997_3**	0.43		0.57		-0.23		0.30		8,199	NA
**1997_4**	0.44		0.58		-0.22		0.30		8,067	NA
**1998_1**	0.43		0.58		-0.21		0.31		8,103	NA
**1998_2**	0.45		0.59		-0.21		0.30		7,913	NA
**1998_3**	0.43		0.59		-0.19		0.30		7,855	NA
**1998_4**	0.45		0.58		-0.21		0.30		8,024	NA
**1999_1**	0.46		0.59		-0.21		0.29		7,977	NA
**1999_2**	0.46		0.58		-0.21		0.29		7,887	NA
**1999_3**	0.45		0.58		-0.20		0.30		7,798	NA
**1999_4**	0.45		0.57		-0.20		0.30		7,883	NA
**2000_1**	0.44		0.57		-0.17		0.29		7,899	NA
**2000_2**	0.44		0.57		-0.20		0.31		7,840	NA
**2000_3**	0.46		0.57		-0.20		0.30		7,795	NA
**2000_4**	0.44		0.58		-0.18		0.30		7,607	NA
**2001_1**	0.44		0.57		-0.19		0.30		7,908	NA
**2001_2**	0.43		0.57		-0.19		0.30		7,903	NA
**2001_3**	0.42		0.57		-0.17		0.31		7,850	NA
**2001_4**	0.44		0.57		-0.16		0.33		8,020	NA
**2002_1**	0.44		0.57		-0.17		0.33		7,909	NA
**2002_2**	0.44		0.58		-0.17		0.33		7,640	NA
**2002_3**	0.42		0.57		-0.17		0.33		7,718	NA
**2002_4**	0.41		0.56		-0.17		0.32		7,732	NA
**2003_1**	0.40		0.55		-0.19		0.31		7,572	NA
**2003_2**	0.41		0.56		-0.20		0.32		7,431	NA
**2003_3**	0.43		0.57		-0.19		0.33		7,535	NA
**2003_4**	0.42		0.56		-0.18		0.32		7,748	NA
**2004_1**	0.40		0.56		-0.18		0.32		7,724	NA
**2004_2**	0.42		0.56		-0.18		0.32		7,662	NA
**2004_3**	0.42		0.55		-0.19		0.33		7,745	NA
**2004_4**	0.39		0.55		-0.19		0.32		7,718	NA
**2005_1**	0.42		0.55		-0.19		0.32		7,792	NA
**2005_2**	0.42		0.55		-0.17		0.31		7,612	NA
**2005_3**	0.42	0.45	0.53	0.59	-0.20	-0.22	0.32	0.40	7,438	1,373
**2005_4**	0.41	0.41	0.53	0.59	-0.19	-0.22	0.31	0.43	7,519	2,699
**2006_1**	0.41	0.45	0.56	0.61	-0.18	-0.20	0.31	0.43	7,476	2,906
**2006_2**	0.43	0.43	0.55	0.60	-0.17	-0.18	0.33	0.39	7,435	5,825
**2006_3**	0.41	0.38	0.56	0.56	-0.18	-0.16	0.34	0.36	7,164	13,428
**2006_4**	0.40	0.41	0.54	0.57	-0.19	-0.17	0.34	0.36	7,149	13,589
**2007_1**	0.39	0.42	0.52	0.57	-0.19	-0.18	0.34	0.37	7,241	13,538
**2007_2**	0.39	0.41	0.50	0.57	-0.20	-0.18	0.35	0.37	7,190	14,337
**2007_3**	0.38	0.42	0.51	0.57	-0.18	-0.17	0.35	0.35	7,077	18,231
**2007_4**	0.37	0.43	0.51	0.57	-0.18	-0.16	0.33	0.35	7,231	21,999
**2008_1**	0.38	0.43	0.50	0.58	-0.19	-0.18	0.33	0.35	7,137	22,307
**2008_2**	0.37	0.43	0.51	0.57	-0.19	-0.18	0.34	0.35	7,097	28,323
**2008_3**	0.37	0.42	0.50	0.58	-0.18	-0.17	0.33	0.34	6,898	30,750
**2008_4**	0.36	0.43	0.50	0.58	-0.21	-0.18	0.35	0.35	7,038	31,373
**2009_1**	0.36	0.42	0.49	0.58	-0.16	-0.17	0.33	0.34	6,928	31,305
**2009_2**	0.36	0.41	0.49	0.58	-0.18	-0.16	0.35	0.34	7,124	40,454
**2009_3**	0.34	0.40	0.49	0.57	-0.18	-0.15	0.35	0.34	6,947	61,281
**2009_4**	0.36	0.40	0.49	0.57	-0.20	-0.15	0.34	0.33	7,192	74,405
**2010_1**	0.36	0.40	0.49	0.57	-0.18	-0.16	0.35	0.34	7,026	77,427
**2010_2**	0.37	0.40	0.49	0.57	-0.17	-0.15	0.34	0.34	6,954	77,554
**2010_3**	0.39	0.41	0.51	0.57	-0.18	-0.16	0.34	0.34	6,979	78,103
**2010_4**	0.37	0.40	0.52	0.57	-0.17	-0.16	0.34	0.33	7,032	78,497
**2011_1**	0.37	0.40	0.53	0.56	-0.19	-0.16	0.33	0.33	6,943	78,255
**Intercept**	0.46	0.43	0.60	0.59	0.21	0.20	0.29	0.40	–	–
**Slope**	0.00	0.00	0.00	0.00	0.00	0.00	0.00	0.00	–	–

*Cramer’s V.

**Pearson’s R.

Table 5 examines patterns of scale reliability over time as, measured using Cronbach’s alpha statistic for internal consistency. Using a cut-off of 0.70 for acceptable reliability and 0.80 for excellent reliability, all three scales (ADL Long Form, DRS, ABS) displayed acceptable or excellent reliability over the entire study period and between the two care settings. The alpha values were lowest for the DRS and highest for the ADL Long Form scale, which is consistent with previous reports in the literature.

**Table 5:** rends in internal consistency (Cronbach’s alpha values) for ADL, depression and aggressive behaviour scales over time, by facility type (CCC and LTC), Ontario

	**ADL long form**		**Depression rating scale**		**Aggressive behaviour scale**		**Number of assessments**	
**Quarter**	**CCC**	**LTC**	**CCC**	**LTC**	**CCC**	**LTC**	**CCC**	**LTC**
**1996_3**	0.91		0.76		0.76		9,164	NA
**1996_4**	0.91		0.76		0.75		8,604	NA
**1997_1**	0.91		0.75		0.76		8,372	NA
**1997_2**	0.92		0.76		0.77		7,776	NA
**1997_3**	0.91		0.77		0.77		8,199	NA
**1997_4**	0.92		0.77		0.77		8,067	NA
**1998_1**	0.91		0.77		0.77		8,103	NA
**1998_2**	0.92		0.76		0.78		7,913	NA
**1998_3**	0.92		0.76		0.78		7,855	NA
**1998_4**	0.92		0.76		0.77		8,024	NA
**1999_1**	0.92		0.76		0.78		7,977	NA
**1999_2**	0.92		0.75		0.78		7,887	NA
**1999_3**	0.92		0.75		0.78		7,798	NA
**1999_4**	0.92		0.75		0.78		7,883	NA
**2000_1**	0.92		0.75		0.79		7,899	NA
**2000_2**	0.92		0.76		0.80		7,840	NA
**2000_3**	0.92		0.75		0.80		7,795	NA
**2000_4**	0.92		0.76		0.80		7,607	NA
**2001_1**	0.92		0.75		0.79		7,908	NA
**2001_2**	0.92		0.75		0.79		7,903	NA
**2001_3**	0.92		0.75		0.78		7,850	NA
**2001_4**	0.92		0.74		0.78		8,020	NA
**2002_1**	0.92		0.74		0.79		7,909	NA
**2002_2**	0.92		0.75		0.79		7,640	NA
**2002_3**	0.92		0.75		0.79		7,718	NA
**2002_4**	0.92		0.75		0.78		7,732	NA
**2003_1**	0.92		0.75		0.79		7,572	NA
**2003_2**	0.92		0.76		0.77		7,431	NA
**2003_3**	0.92		0.77		0.78		7,535	NA
**2003_4**	0.92		0.76		0.77		7,748	NA
**2004_1**	0.92		0.76		0.77		7,724	NA
**2004_2**	0.92		0.75		0.78		7,662	NA
**2004_3**	0.92		0.76		0.77		7,745	NA
**2004_4**	0.92		0.76		0.76		7,718	NA
**2005_1**	0.92		0.76		0.77		7,792	NA
**2005_2**	0.92		0.75		0.77		7,612	NA
**2005_3**	0.92	0.92	0.75	0.72	0.78	0.79	7,438	1,373
**2005_4**	0.93	0.93	0.75	0.70	0.79	0.75	7,519	2,699
**2006_1**	0.93	0.93	0.77	0.73	0.78	0.75	7,476	2,906
**2006_2**	0.93	0.93	0.77	0.71	0.79	0.74	7,435	5,825
**2006_3**	0.93	0.93	0.76	0.71	0.80	0.73	7,164	13,428
**2006_4**	0.93	0.93	0.76	0.70	0.80	0.72	7,149	13,589
**2007_1**	0.92	0.93	0.75	0.70	0.80	0.72	7,241	13,538
**2007_2**	0.92	0.93	0.76	0.70	0.80	0.72	7,190	14,337
**2007_3**	0.92	0.94	0.76	0.70	0.80	0.72	7,077	18,231
**2007_4**	0.92	0.94	0.76	0.71	0.80	0.73	7,231	21,999
**2008_1**	0.92	0.94	0.76	0.71	0.80	0.73	7,137	22,307
**2008_2**	0.92	0.94	0.75	0.71	0.79	0.73	7,097	28,323
**2008_3**	0.92	0.94	0.76	0.71	0.80	0.73	6,898	30,750
**2008_4**	0.92	0.94	0.76	0.72	0.79	0.72	7,038	31,373
**2009_1**	0.92	0.94	0.75	0.71	0.81	0.72	6,928	31,305
**2009_2**	0.92	0.94	0.75	0.72	0.80	0.73	7,124	40,454
**2009_3**	0.92	0.94	0.75	0.72	0.80	0.73	6,947	61,281
**2009_4**	0.92	0.94	0.75	0.71	0.81	0.73	7,192	74,405
**2010_1**	0.92	0.94	0.75	0.71	0.79	0.73	7,026	77,427
**2010_2**	0.92	0.94	0.74	0.71	0.80	0.74	6,954	77,554
**2010_3**	0.92	0.94	0.75	0.71	0.80	0.74	6,979	78,103
**2010_4**	0.93	0.94	0.75	0.71	0.79	0.73	7,032	78,497
**2011_1**	0.92	0.94	0.73	0.71	0.79	0.73	6,943	78,255
**Intercept**	0.92	0.93	0.76	0.71	0.77	0.74	–	–
**Slope**	0.00	0.00	0.00	0.00	0.00	-0.00	–	–

The following three tables consider cross-sectional logical inconsistency in coding of clinical and service utilization variables. Table 6 shows the rate of the following logical errors in coding:

•mood persistence - the mood persistence item records mood indicators are present, but none of the individual items for mood indicators are coded as being present

•ADL – one, but not both, of the ratings for support and performance of specific ADLs are coded as “8 – did not occur”

•parental/enteral intake - a mismatch between being reported to have parenteral/IV or feeding tube present and the proportion of total calories and fluid intake from those sources per day), and

•pressure ulcer staging (i.e., a “highest stage” value is assigned for pressure or stasis ulcers but the number of ulcers at that stage is missing or equal to zero).

**Table 6:** Trends in logical inconsistencies in selected clinical characteristics over time, by facility type (CCC and LTC), Ontario

	**Mood persistence but no mood item**		**ADL did not occur inconsistency**		**Parenteral/enteral intake inconsistency**		**Ulcer staging inconsistency**		**Number of assessments**	
**Quarter**	**CCC**	**LTC**	**CCC**	**LTC**	**CCC**	**LTC**	**CCC**	**LTC**	**CCC**	**LTC**
**1996_3**	1.1%		2.3%		0.1%		1.2%		9,164	NA
**1996_4**	1.2%		2.1%		0.9%		1.0%		8,604	NA
**1997_1**	0.9%		1.7%		1.0%		1.1%		8,372	NA
**1997_2**	0.8%		2.0%		1.6%		1.1%		7,776	NA
**1997_3**	1.0%		1.5%		0.9%		1.3%		8,199	NA
**1997_4**	1.3%		1.2%		0.9%		1.3%		8,067	NA
**1998_1**	1.0%		1.2%		1.0%		1.4%		8,103	NA
**1998_2**	0.9%		1.5%		1.0%		1.6%		7,913	NA
**1998_3**	1.0%		1.5%		1.0%		2.5%		7,855	NA
**1998_4**	0.8%		1.3%		0.9%		2.8%		8,024	NA
**1999_1**	0.8%		1.3%		0.8%		2.8%		7,977	NA
**1999_2**	0.8%		1.5%		1.1%		2.9%		7,887	NA
**1999_3**	0.7%		1.5%		0.9%		2.7%		7,798	NA
**1999_4**	0.8%		1.4%		1.0%		2.8%		7,883	NA
**2000_1**	0.7%		1.2%		1.1%		2.8%		7,899	NA
**2000_2**	0.7%		1.3%		0.1%		3.1%		7,840	NA
**2000_3**	0.7%		1.3%		0.3%		2.9%		7,795	NA
**2000_4**	0.9%		1.1%		0.1%		2.7%		7,607	NA
**2001_1**	0.8%		1.0%		0.1%		2.5%		7,908	NA
**2001_2**	0.7%		1.0%		0.1%		2.2%		7,903	NA
**2001_3**	0.7%		0.8%		0.0%		2.0%		7,850	NA
**2001_4**	0.7%		0.9%		0.1%		2.0%		8,020	NA
**2002_1**	0.6%		0.7%		0.1%		2.2%		7,909	NA
**2002_2**	0.6%		0.6%		0.1%		2.3%		7,640	NA
**2002_3**	0.6%		0.6%		0.1%		2.3%		7,718	NA
**2002_4**	0.4%		0.7%		0.0%		2.1%		7,732	NA
**2003_1**	0.5%		0.6%		0.1%		2.2%		7,572	NA
**2003_2**	0.3%		0.2%		0.0%		0.9%		7,431	NA
**2003_3**	0.0%		0.0%		0.0%		0.1%		7,535	NA
**2003_4**	0.0%		0.0%		0.0%		0.0%		7,748	NA
**2004_1**	0.0%		0.0%		0.0%		0.0%		7,724	NA
**2004_2**	0.0%		0.0%		0.0%		0.0%		7,662	NA
**2004_3**	0.0%		0.0%		0.0%		0.0%		7,745	NA
**2004_4**	0.0%		0.0%		0.0%		0.0%		7,718	NA
**2005_1**	0.0%		0.0%		0.0%		0.0%		7,792	NA
**2005_2**	0.0%		0.0%		0.0%		0.0%		7,612	NA
**2005_3**	0.0%	0.0%	0.0%	0.0%	0.0%	0.0%	0.0%	0.0%	7,438	1,373
**2005_4**	0.0%	0.0%	0.0%	0.0%	0.0%	0.0%	0.0%	0.0%	7,519	2,699
**2006_1**	0.0%	0.0%	0.0%	0.0%	0.0%	0.0%	0.0%	0.0%	7,476	2,906
**2006_2**	0.0%	0.0%	0.0%	0.0%	0.0%	0.0%	0.0%	0.0%	7,435	5,825
**2006_3**	0.0%	0.0%	0.0%	0.0%	0.0%	0.0%	0.0%	0.0%	7,164	13,428
**2006_4**	0.0%	0.0%	0.0%	0.0%	0.0%	0.0%	0.0%	0.0%	7,149	13,589
**2007_1**	0.0%	0.0%	0.0%	0.0%	0.0%	0.0%	0.0%	0.0%	7,241	13,538
**2007_2**	0.0%	0.0%	0.0%	0.0%	0.0%	0.0%	0.0%	0.0%	7,190	14,337
**2007_3**	0.0%	0.0%	0.0%	0.0%	0.0%	0.0%	0.0%	0.0%	7,077	18,231
**2007_4**	0.0%	0.0%	0.0%	0.0%	0.0%	0.0%	0.0%	0.0%	7,231	21,999
**2008_1**	0.0%	0.0%	0.0%	0.0%	0.0%	0.0%	0.0%	0.0%	7,137	22,307
**2008_2**	0.0%	0.0%	0.0%	0.0%	0.0%	0.0%	0.0%	0.0%	7,097	28,323
**2008_3**	0.0%	0.0%	0.0%	0.0%	0.0%	0.0%	0.0%	0.0%	6,898	30,750
**2008_4**	0.0%	0.0%	0.0%	0.0%	0.0%	0.0%	0.0%	0.0%	7,038	31,373
**2009_1**	0.0%	0.0%	0.0%	0.0%	0.0%	0.0%	0.0%	0.0%	6,928	31,305
**2009_2**	0.0%	0.0%	0.0%	0.0%	0.0%	0.0%	0.0%	0.0%	7,124	40,454
**2009_3**	0.0%	0.0%	0.0%	0.0%	0.0%	0.0%	0.0%	0.0%	6,947	61,281
**2009_4**	0.0%	0.0%	0.0%	0.0%	0.0%	0.0%	0.0%	0.0%	7,192	74,405
**2010_1**	0.0%	0.0%	0.0%	0.0%	0.0%	0.0%	0.0%	0.0%	7,026	77,427
**2010_2**	0.0%	0.0%	0.0%	0.0%	0.0%	0.0%	0.0%	0.0%	6,954	77,554
**2010_3**	0.0%	0.0%	0.0%	0.0%	0.0%	0.0%	0.0%	0.0%	6,979	78,103
**2010_4**	0.0%	0.0%	0.0%	0.0%	0.0%	0.0%	0.0%	0.0%	7,032	78,497
**2011_1**	0.0%	0.0%	0.0%	0.0%	0.0%	0.0%	0.0%	0.0%	6,943	78,255
**Intercept**	1.1%	0.0%	1.7%	0.0%	0.8%	0.0%	2.5%	0.0%	–	–
**Slope**	-0.02%	0.00%	-0.04%	0.00%	-0.02%	0.00%	-0.05%	0.00%	–	–

These logical errors occurred at very low rates when the RAI 2.0 was originally mandated in CCC. Rates were below 5% at the outset, except for the inconsistency for parenteral/enteral intake in CCC which was below 10% in the initial years of implementation. However, they were completely eliminated in CCC after 2003 when CIHI began to use data quality checks for such errors in its acceptance procedures. After that date, data submissions with these errors present were rejected by CCRS and hospitals were required to remediate the problems prior to resubmissions. These errors never appeared in the LTC data, which is unsurprising given that implementation in that sector occurred after the change was made to CCRS regarding those errors.

As with the abovementioned clinical logical error checks, the rates of logical errors related to coding of therapy time was very low over the entire study period (see Table 7). By 2011, the only logical error that persisted was for therapy of less than 15 minutes being counted as a day with therapy, which was present for about 2% of cases.

**Table 7:** Trends in logical inconsistencies in therapy coding over time, by facility type (CCC and LTC), Ontario

	**Therapy problem: 15 min/day counted as day**		**Therapy problem: no days when sufficient minutes for at least 1 day**		**Therapy problem: minutes exceed minutes in a day**		**Any of the three therapy problems**		**Any logical problem (mood, ADL, feed, ulcer, therapy)**		**Number of assessments**	
**Quarter**	**CCC**	**LTC**	**CCC**	**LTC**	**CCC**	**LTC**	**CCC**	**LTC**	**CCC**	**LTC**	**CCC**	**LTC**
**1996_3**	1.6%		0.1%		0.0%		1.7%		6.4%		9,164	NA
**1996_4**	1.9%		0.0%		0.0%		2.0%		7.1%		8,604	NA
**1997_1**	2.0%		0.0%		0.0%		2.1%		6.8%		8,372	NA
**1997_2**	2.1%		0.1%		0.1%		2.3%		7.8%		7,776	NA
**1997_3**	2.0%		0.1%		0.0%		2.0%		6.8%		8,199	NA
**1997_4**	1.7%		0.0%		0.0%		1.8%		6.5%		8,067	NA
**1998_1**	2.2%		0.0%		0.0%		2.3%		7.0%		8,103	NA
**1998_2**	2.0%		0.2%		0.1%		2.2%		7.2%		7,913	NA
**1998_3**	2.1%		0.1%		0.0%		2.2%		8.2%		7,855	NA
**1998_4**	1.7%		0.1%		0.0%		1.8%		7.6%		8,024	NA
**1999_1**	2.0%		0.1%		0.2%		2.2%		8.0%		7,977	NA
**1999_2**	1.9%		0.1%		0.1%		2.0%		8.2%		7,887	NA
**1999_3**	1.6%		0.1%		0.0%		1.7%		7.5%		7,798	NA
**1999_4**	2.0%		0.0%		0.1%		2.1%		8.1%		7,883	NA
**2000_1**	1.6%		0.1%		0.1%		1.8%		7.7%		7,899	NA
**2000_2**	1.4%		0.1%		0.0%		1.5%		6.7%		7,840	NA
**2000_3**	1.3%		0.0%		0.1%		1.3%		6.4%		7,795	NA
**2000_4**	1.7%		0.1%		0.1%		1.9%		6.7%		7,607	NA
**2001_1**	1.3%		0.0%		0.0%		1.4%		5.7%		7,908	NA
**2001_2**	1.3%		0.1%		0.1%		1.5%		5.5%		7,903	NA
**2001_3**	1.7%		0.1%		0.1%		1.9%		5.3%		7,850	NA
**2001_4**	1.5%		0.0%		0.0%		1.6%		5.2%		8,020	NA
**2002_1**	1.3%		0.1%		0.0%		1.4%		4.9%		7,909	NA
**2002_2**	1.3%		0.1%		0.1%		1.5%		5.2%		7,640	NA
**2002_3**	1.2%		0.1%		0.0%		1.3%		4.9%		7,718	NA
**2002_4**	0.8%		0.1%		0.0%		0.9%		4.2%		7,732	NA
**2003_1**	0.8%		0.0%		0.0%		0.9%		4.2%		7,572	NA
**2003_2**	1.1%		0.1%		0.0%		1.3%		2.7%		7,431	NA
**2003_3**	1.9%		0.0%		0.0%		1.9%		2.1%		7,535	NA
**2003_4**	2.2%		0.0%		0.0%		2.2%		2.2%		7,748	NA
**2004_1**	1.4%		0.0%		0.0%		1.4%		1.4%		7,724	NA
**2004_2**	1.5%		0.0%		0.0%		1.5%		1.5%		7,662	NA
**2004_3**	2.5%		0.0%		0.0%		2.6%		2.6%		7,745	NA
**2004_4**	2.0%		0.0%		0.0%		2.0%		2.0%		7,718	NA
**2005_1**	2.2%		0.0%		0.1%		2.2%		2.2%		7,792	NA
**2005_2**	2.3%		0.0%		0.0%		2.3%		2.3%		7,612	NA
**2005_3**	2.6%	3.0%	0.0%	0.0%	0.0%	0.0%	2.6%	3.0%	2.6%	3.0%	7,438	1,373
**2005_4**	2.6%	1.9%	0.0%	0.0%	0.0%	0.0%	2.6%	1.9%	2.6%	1.9%	7,519	2,699
**2006_1**	2.9%	2.2%	0.0%	0.0%	0.0%	0.0%	2.9%	2.2%	2.9%	2.2%	7,476	2,906
**2006_2**	2.5%	2.5%	0.0%	0.0%	0.1%	0.0%	2.6%	2.5%	2.6%	2.5%	7,435	5,825
**2006_3**	2.5%	2.4%	0.0%	0.0%	0.0%	0.0%	2.6%	2.5%	2.6%	2.5%	7,164	13,428
**2006_4**	2.5%	2.5%	0.0%	0.0%	0.0%	0.0%	2.6%	2.5%	2.6%	2.5%	7,149	13,589
**2007_1**	2.5%	2.5%	0.0%	0.0%	0.0%	0.1%	2.5%	2.6%	2.5%	2.6%	7,241	13,538
**2007_2**	2.3%	2.8%	0.0%	0.0%	0.0%	0.0%	2.3%	2.8%	2.3%	2.8%	7,190	14,337
**2007_3**	2.7%	2.4%	0.0%	0.0%	0.0%	0.0%	2.8%	2.4%	2.8%	2.4%	7,077	18,231
**2007_4**	2.1%	2.4%	0.0%	0.0%	0.0%	0.0%	2.2%	2.5%	2.2%	2.5%	7,231	21,999
**2008_1**	2.2%	2.7%	0.0%	0.0%	0.0%	0.0%	2.2%	2.7%	2.2%	2.7%	7,137	22,307
**2008_2**	2.5%	2.7%	0.0%	0.0%	0.0%	0.0%	2.5%	2.7%	2.5%	2.7%	7,097	28,323
**2008_3**	2.3%	2.5%	0.0%	0.0%	0.0%	0.0%	2.3%	2.5%	2.3%	2.5%	6,898	30,750
**2008_4**	2.5%	2.4%	0.0%	0.0%	0.0%	0.0%	2.5%	2.4%	2.5%	2.4%	7,038	31,373
**2009_1**	2.4%	2.9%	0.0%	0.0%	0.0%	0.0%	2.5%	2.9%	2.5%	2.9%	6,928	31,305
**2009_2**	2.8%	2.6%	0.0%	0.0%	0.0%	0.0%	2.8%	2.6%	2.8%	2.6%	7,124	40,454
**2009_3**	2.7%	2.2%	0.0%	0.0%	0.0%	0.0%	2.7%	2.2%	2.7%	2.2%	6,947	61,281
**2009_4**	2.0%	1.9%	0.0%	0.0%	0.0%	0.0%	2.0%	1.9%	2.0%	1.9%	7,192	74,405
**2010_1**	2.2%	1.6%	0.0%	0.0%	0.0%	0.0%	2.2%	1.6%	2.2%	1.6%	7,026	77,427
**2010_2**	2.1%	1.5%	0.0%	0.0%	0.0%	0.0%	2.1%	1.5%	2.1%	1.5%	6,954	77,554
**2010_3**	2.0%	1.3%	0.0%	0.0%	0.0%	0.0%	2.0%	1.4%	2.0%	1.4%	6,979	78,103
**2010_4**	2.0%	1.4%	0.0%	0.0%	0.0%	0.0%	2.0%	1.4%	2.0%	1.4%	7,032	78,497
**2011_1**	2.3%	1.4%	0.0%	0.0%	0.0%	0.0%	2.3%	1.4%	2.3%	1.4%	6,943	78,255
**Intercept**	1.6%	2.8%	0.1%	0.0%	0.1%	0.0%	1.7%	2.9%	7.7%	2.9%	–	–
**Slope**	0.01%	-0.05%	-0.00%	0.00%	-0.00%	-0.00%	0.01%	-0.05%	-0.12%	-0.05%	–	–

A similar pattern is evident in Table 8, which shows very low rates of probable error in coding height and age (both with rates below 5%), although CCC hospitals/units were more likely than LTC to have problems with coding height. The rates of logical errors in coding weight were considerably higher in CCC between 1996 and 2003, with rates as high as 21%. However, for both settings these rates fell to 5% or lower in the last year of the study period, and in LTC problems with coding of weight were evident less than 1% of the time.

**Table 8:** Trends in logical inconsistencies in height, weight and age coding over time, by facility type (CCC and LTC), Ontario

	**Height (non-missing and <120 cm or >211 cm)**		**Weight (non-missing and < 20 kg or >200 kg)**		**Age at assessment <16 or >115 years**		**Frequency**	
**Quarter**	**CCC**	**LTC**	**CCC**	**LTC**	**CCC**	**LTC**	**CCC**	**LTC**
**1996_3**	2.1%		9.3%		0.0%		9,164	NA
**1996_4**	1.8%		11.7%		0.2%		8,604	NA
**1997_1**	2.4%		13.3%		0.2%		8,372	NA
**1997_2**	2.7%		16.0%		0.3%		7,776	NA
**1997_3**	2.4%		19.4%		0.3%		8,199	NA
**1997_4**	2.4%		20.6%		0.2%		8,067	NA
**1998_1**	2.3%		20.5%		0.2%		8,103	NA
**1998_2**	2.5%		20.7%		0.3%		7,913	NA
**1998_3**	2.5%		19.5%		0.3%		7,855	NA
**1998_4**	2.7%		18.9%		0.3%		8,024	NA
**1999_1**	3.1%		16.0%		0.2%		7,977	NA
**1999_2**	3.0%		17.0%		0.3%		7,887	NA
**1999_3**	3.1%		15.8%		0.3%		7,798	NA
**1999_4**	3.5%		13.1%		0.2%		7,883	NA
**2000_1**	3.6%		12.4%		0.2%		7,899	NA
**2000_2**	3.8%		10.4%		0.2%		7,840	NA
**2000_3**	3.6%		10.1%		0.2%		7,795	NA
**2000_4**	3.9%		11.2%		0.2%		7,607	NA
**2001_1**	4.3%		11.1%		0.2%		7,908	NA
**2001_2**	4.6%		10.9%		0.2%		7,903	NA
**2001_3**	4.9%		10.1%		0.2%		7,850	NA
**2001_4**	4.3%		10.0%		0.2%		8,020	NA
**2002_1**	3.5%		10.6%		0.3%		7,909	NA
**2002_2**	3.2%		12.0%		0.3%		7,640	NA
**2002_3**	3.6%		11.9%		0.3%		7,718	NA
**2002_4**	3.8%		11.4%		0.3%		7,732	NA
**2003_1**	4.2%		10.7%		0.3%		7,572	NA
**2003_2**	4.4%		5.4%		0.3%		7,431	NA
**2003_3**	4.8%		5.0%		0.4%		7,535	NA
**2003_4**	3.6%		4.5%		0.3%		7,748	NA
**2004_1**	3.7%		3.5%		0.3%		7,724	NA
**2004_2**	3.4%		3.1%		0.3%		7,662	NA
**2004_3**	3.4%		3.6%		0.4%		7,745	NA
**2004_4**	3.1%		3.4%		0.3%		7,718	NA
**2005_1**	2.8%		3.4%		0.3%		7,792	NA
**2005_2**	3.5%		3.6%		0.3%		7,612	NA
**2005_3**	3.7%	0.3%	3.9%	0.1%	0.3%	0.0%	7,438	1,373
**2005_4**	3.7%	0.5%	4.0%	0.1%	0.5%	0.0%	7,519	2,699
**2006_1**	4.1%	0.5%	4.1%	0.1%	0.4%	0.0%	7,476	2,906
**2006_2**	4.2%	0.5%	4.7%	0.1%	0.3%	0.0%	7,435	5,825
**2006_3**	5.0%	0.6%	4.7%	0.3%	0.3%	0.0%	7,164	13,428
**2006_4**	4.6%	0.5%	4.5%	0.2%	0.2%	0.0%	7,149	13,589
**2007_1**	4.5%	0.5%	4.3%	0.2%	0.3%	0.0%	7,241	13,538
**2007_2**	5.3%	0.6%	4.3%	0.3%	0.4%	0.0%	7,190	14,337
**2007_3**	5.5%	0.5%	4.6%	0.3%	0.3%	0.0%	7,077	18,231
**2007_4**	5.7%	0.6%	4.9%	0.4%	0.3%	0.0%	7,231	21,999
**2008_1**	4.8%	0.6%	4.5%	0.2%	0.3%	0.0%	7,137	22,307
**2008_2**	4.7%	0.4%	4.5%	0.2%	0.3%	0.0%	7,097	28,323
**2008_3**	4.5%	0.3%	4.3%	0.2%	0.3%	0.0%	6,898	30,750
**2008_4**	3.7%	0.3%	3.4%	0.2%	0.3%	0.0%	7,038	31,373
**2009_1**	3.9%	0.3%	4.0%	0.3%	0.3%	0.0%	6,928	31,305
**2009_2**	4.1%	0.4%	4.2%	0.2%	0.2%	0.0%	7,124	40,454
**2009_3**	4.3%	0.4%	3.9%	0.2%	0.3%	0.0%	6,947	61,281
**2009_4**	3.9%	0.3%	3.9%	0.3%	0.3%	0.0%	7,192	74,405
**2010_1**	4.5%	0.3%	4.4%	0.3%	0.2%	0.0%	7,026	77,427
**2010_2**	3.9%	0.3%	4.1%	0.3%	0.2%	0.0%	6,954	77,554
**2010_3**	4.5%	0.3%	4.3%	0.3%	0.2%	0.0%	6,979	78,103
**2010_4**	4.1%	0.3%	3.9%	0.3%	0.2%	0.0%	7,032	78,497
**2011_1**	4.1%	0.3%	4.1%	0.3%	0.2%	0.0%	6,943	78,255
**Intercept**	2.7%	0.5%	16.7%	0.2%	0.2%	0.0%	–	–
**Slope**	0.04%	-0.01%	-0.27%	0.01%	0.00%	0.00%	–	–

**Figure 1:**
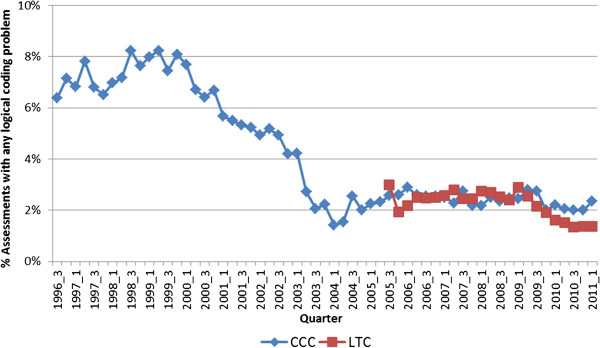
Trends in the rate of any logical coding problem over time by setting, Ontario, 1996-2011.

Figure 1 shows that the rate for any logical problem in coding of mood indicators, ADL, nutritional intake, pressure ulcers, and therapy were below 10% for the entire study period. There was also a clear trend toward improved data quality in these areas over time. Both the CCC and LTC settings had steady rates of approximately 2% for these indicators after 2005.

The next set of analyses uses longitudinal records to identify unlikely reversals in chronic diseases coded at the initial assessment (see Table 9). If either multiple sclerosis, quadriplegia, cerebral palsy or schizophrenia are noted on an initial assessment, it would be unusual for the condition to have been reversed by the follow-up assessment. Although the symptoms of multiple sclerosis can be less pronounced at times, none of these conditions is considered curable. Either the initial assessment was inaccurate (for which a correction should have been submitted) or this diagnosis was incorrectly coded as absent at follow-up. The instances of not having the condition at follow-up among those who had it coded at the initial assessment became increasingly uncommon over time. However, in the initial phases of implementation about one fifth of those with quadriplegia or with cerebral palsy had this error at follow up. While these problems appear to be declining over time, it would be fairly easy to eliminate them with appropriate longitudinal error checks.

**Table 9:** Trends in improbable patterns in diagnosis between initial (T1) and follow-up (T2) assessments, by facility type (CCC and LTC), Ontario

	**Multiple sclerosis recorded at T1 but not at T2**		**Quadriplegia recorded at T1 but not at T2**		**Cerebral palsy recorded at T1 but not at T2**		**Schizophrenia recorded at T1 but not at T2**		**Number of assessments**	
**Quarter**	**CCC**	**LTC**	**CCC**	**LTC**	**CCC**	**LTC**	**CCC**	**LTC**	**CCC**	**LTC**
**1996_3**	5.5%		17.5%		14.9%				3,853	NA
**1996_4**	4.2%		19.2%		16.0%				3,600	NA
**1997_1**	9.2%		20.6%		9.4%				3,213	NA
**1997_2**	5.0%		24.4%		8.6%				3,184	NA
**1997_3**	6.4%		21.0%		14.7%				3,331	NA
**1997_4**	5.0%		19.8%		23.0%				3,329	NA
**1998_2**	4.9%		18.2%		17.2%				3,292	NA
**1998_3**	5.1%		13.0%		9.5%				3,290	NA
**1998_4**	8.2%		21.7%		19.1%				3,259	NA
**1999_1**	3.1%		17.1%		19.4%				3,131	NA
**1999_2**	4.0%		12.2%		8.2%				3,172	NA
**1999_3**	5.6%		13.3%		13.6%				3,242	NA
**1999_4**	6.5%		20.9%		16.9%				3,138	NA
**2000_1**	7.5%		18.1%		11.7%				3,104	NA
**2000_2**	4.5%		17.5%		17.7%				3,080	NA
**2000_3**	4.0%		18.5%		15.2%				3,133	NA
**2000_4**	6.3%		15.6%		15.4%				2,961	NA
**2001_1**	3.7%		16.1%		11.1%				2,915	NA
**2001_2**	5.1%		11.3%		11.6%				2,965	NA
**2001_3**	4.2%		12.3%		9.5%				3,059	NA
**2001_4**	5.5%		19.1%		10.3%				3,103	NA
**2002_1**	6.0%		13.9%		3.8%				3,168	NA
**2002_2**	3.5%		10.7%		10.3%				3,030	NA
**2002_3**	4.4%		14.9%		14.5%				3,041	NA
**2002_4**	5.5%		13.3%		15.2%				3,042	NA
**2003_1**	5.8%		8.4%		14.7%				2,931	NA
**2003_2**	3.3%		19.7%		11.3%		38.1%		2,808	NA
**2003_3**	4.2%		9.0%		13.3%		16.7%		2,726	NA
**2003_4**	2.5%		10.9%		11.1%		10.3%		2,720	NA
**2004_1**	2.4%		12.3%		16.4%		7.2%		2,715	NA
**2004_2**	1.5%		8.9%		4.7%		5.4%		2,722	NA
**2004_3**	0.5%		11.7%		4.8%		2.9%		2,738	NA
**2004_4**	2.1%		4.4%		9.1%		1.4%		2,657	NA
**2005_1**	0.0%		7.0%		1.8%		6.8%		2,631	NA
**2005_2**	1.1%		7.7%		8.3%		10.8%		2,552	NA
**2005_3**	1.1%		6.3%		3.6%		7.0%		2,503	NA
**2005_4**	2.4%	33.3%	5.3%	0.0%	5.6%	16.7%	11.5%	22.2%	2,500	880
**2006_1**	0.6%	0.0%	4.0%	0.0%	7.8%	11.1%	3.6%	4.3%	2,461	1,941
**2006_2**	2.6%	0.0%	2.2%	20.0%	7.1%	5.9%	5.8%	8.0%	2,478	1,960
**2006_3**	2.5%	5.0%	8.5%	5.6%	5.3%	5.7%	3.9%	4.2%	2,541	4,209
**2006_4**	1.8%	4.1%	5.1%	0.0%	0.0%	5.6%	3.2%	4.1%	2,516	9,708
**2007_1**	1.8%	1.0%	4.9%	2.1%	13.3%	5.3%	3.6%	1.8%	2,480	9,699
**2007_2**	0.0%	0.9%	2.9%	7.3%	0.0%	4.4%	0.0%	3.0%	2,457	9,732
**2007_3**	2.7%	4.3%	4.2%	6.0%	2.1%	4.1%	4.7%	4.2%	2,483	10,262
**2007_4**	0.7%	2.5%	3.9%	16.1%	0.0%	11.1%	3.1%	1.7%	2,500	12,887
**2008_1**	1.4%	1.7%	3.9%	5.5%	0.0%	6.1%	8.8%	4.2%	2,479	15,044
**2008_2**	1.5%	3.4%	5.0%	7.3%	6.4%	5.3%	3.1%	2.3%	2,404	15,056
**2008_3**	2.1%	2.1%	1.8%	3.3%	4.3%	3.6%	1.4%	3.0%	2,469	19,116
**2008_4**	0.0%	5.5%	4.5%	6.3%	0.0%	4.3%	5.7%	3.6%	2,424	20,291
**2009_1**	1.4%	1.6%	2.8%	10.2%	2.2%	1.8%	3.1%	4.2%	2,394	20,075
**2009_2**	1.5%	1.2%	5.0%	18.0%	2.3%	4.3%	6.8%	3.5%	2,302	19,721
**2009_3**	0.0%	1.6%	3.3%	8.1%	7.3%	3.8%	5.3%	2.5%	2,282	26,965
**2009_4**	2.5%	3.3%	5.4%	16.0%	2.6%	4.0%	4.5%	3.0%	2,288	42,271
**2010_1**	3.4%	1.6%	3.9%	20.1%	7.0%	4.5%	7.6%	3.1%	2,237	51,059
**2010_2**	2.6%	1.6%	5.1%	13.8%	2.6%	5.6%	6.0%	3.0%	2,250	52,614
**2010_3**	3.9%	1.4%	7.6%	10.3%	4.9%	3.6%	3.1%	2.8%	2,243	52,750
**2010_4**	1.0%	1.8%	6.6%	6.8%	5.6%	3.6%	7.7%	2.8%	2,105	52,064
**2011_1**	0.0%	2.2%	3.5%	7.8%	0.0%	2.7%	6.5%	2.2%	2,021	51,771
**Intercept**	6.3%	8.0%	20.6%	3.9%	16.8%	9.3%	11.7%	7.9%	–	–
**Slope**	-0.10%	-0.38%	-0.33%	0.41%	-0.26%	-0.32%	-0.28%	-0.31%	–	–
				**Total number of consecutive assessment pairs:**					**161,652**	**500,075**

The problem of autopopulation is the focus of analyses reported in Table 10. In an extreme case, autopopulation would be suggested by assessments for which all 231 clinical variables were unchanged between assessments. This is a rare event, although there are some quarters where it has occurred for about 2-3% of patients in CCC facilities. Similar analyses for the 16 mood items and for the 20 ADL self-performance and support items found rates of no change in these values from a previous fiscal quarter in about 35% of CCC patients at the start of the RAI 2.0 mandate, rising to about 47% by 2011. At least some of this stability is reflective of the true absence of clinical change; however, it is interesting to note a much lower rate of identical mood items on reassessment in LTC. When considering the ADL performance opposite trends in potential autopopulation are evident between the two sectors with the proportion having identical scores on 20 ADL items increasing in CCC but decreasing in LTC.

**Table 10:** Trends in assessments with indicators of possible auto-population over time, by facility type (CCC and LTC), Ontario

	All 231 clinical quarterly variables same at reassessment*		All 16 mood items same at reassessment**		All 20 ADL performance and support items same at reassessment*		**Number of assessments**	
**Quarter**	**CCC**	**LTC**	**CCC**	**LTC**	**CCC**	**LTC**	**CCC**	**LTC**
**1996_3**	0.7%		34.8%		34.2%		3,853	NA
**1996_4**	2.2%		35.3%		36.0%		3,600	NA
**1997_1**	1.4%		32.2%		33.5%		3,213	NA
**1997_2**	1.1%		26.4%		29.8%		3,184	NA
**1997_3**	1.0%		32.7%		34.4%		3,331	NA
**1997_4**	1.7%		36.7%		39.1%		3,329	NA
**1998_2**	1.2%		36.8%		38.9%		3,292	NA
**1998_3**	1.0%		33.6%		34.9%		3,290	NA
**1998_4**	0.9%		34.2%		36.1%		3,259	NA
**1999_1**	0.7%		38.1%		41.4%		3,131	NA
**1999_2**	1.1%		38.0%		41.4%		3,172	NA
**1999_3**	0.9%		35.4%		37.7%		3,242	NA
**1999_4**	1.0%		37.1%		39.2%		3,138	NA
**2000_1**	1.0%		35.8%		37.6%		3,104	NA
**2000_2**	0.5%		35.8%		37.8%		3,080	NA
**2000_3**	0.9%		34.6%		38.7%		3,133	NA
**2000_4**	1.2%		35.4%		38.3%		2,961	NA
**2001_1**	1.3%		35.3%		37.9%		2,915	NA
**2001_2**	1.3%		33.2%		35.4%		2,965	NA
**2001_3**	1.2%		34.4%		37.8%		3,059	NA
**2001_4**	0.4%		35.9%		38.2%		3,103	NA
**2002_1**	0.7%		35.7%		37.4%		3,168	NA
**2002_2**	0.3%		35.5%		38.1%		3,030	NA
**2002_3**	0.5%		36.9%		37.6%		3,041	NA
**2002_4**	0.5%		38.2%		39.5%		3,042	NA
**2003_1**	0.4%		41.5%		39.8%		2,931	NA
**2003_2**	0.4%		31.2%		33.5%		2,808	NA
**2003_3**	1.0%		36.8%		38.2%		2,726	NA
**2003_4**	2.4%		41.5%		45.1%		2,720	NA
**2004_1**	1.7%		43.1%		45.9%		2,715	NA
**2004_2**	1.1%		42.5%		44.6%		2,722	NA
**2004_3**	0.8%		44.4%		45.4%		2,738	NA
**2004_4**	1.1%		45.8%		45.5%		2,657	NA
**2005_1**	0.7%		46.0%		47.5%		2,631	NA
**2005_2**	0.7%		44.8%		45.9%		2,552	NA
**2005_3**	0.8%		43.3%		44.9%		2,503	NA
**2005_4**	0.6%	0.5%	45.8%	28.6%	49.0%	39.3%	2,500	880
**2006_1**	0.4%	0.0%	43.7%	15.4%	44.4%	26.0%	2,461	1,941
**2006_2**	0.6%	0.2%	47.0%	16.6%	48.1%	28.3%	2,478	1,960
**2006_3**	0.7%	0.2%	48.6%	20.0%	51.2%	31.2%	2,541	4,209
**2006_4**	0.6%	0.3%	47.7%	19.3%	51.2%	30.5%	2,516	9,708
**2007_1**	0.7%	0.3%	50.0%	18.9%	52.5%	29.8%	2,480	9,699
**2007_2**	0.9%	0.2%	50.0%	18.8%	53.0%	30.1%	2,457	9,732
**2007_3**	0.6%	0.1%	50.6%	20.4%	51.5%	32.4%	2,483	10,262
**2007_4**	2.0%	0.1%	46.4%	22.0%	49.8%	32.5%	2,500	12,887
**2008_1**	1.2%	0.2%	47.2%	22.4%	48.8%	33.9%	2,479	15,044
**2008_2**	1.6%	0.1%	47.7%	24.9%	49.6%	35.0%	2,404	15,056
**2008_3**	0.9%	0.1%	48.6%	25.5%	48.5%	34.9%	2,469	19,116
**2008_4**	0.7%	0.2%	48.1%	26.1%	51.2%	34.4%	2,424	20,291
**2009_1**	0.3%	0.2%	48.8%	26.9%	49.3%	36.1%	2,394	20,075
**2009_2**	1.2%	0.2%	48.1%	28.2%	50.1%	36.3%	2,302	19,721
**2009_3**	0.7%	0.1%	48.0%	24.7%	48.9%	31.2%	2,282	26,965
**2009_4**	0.6%	0.1%	46.6%	20.6%	49.0%	25.9%	2,288	42,271
**2010_1**	0.5%	0.1%	44.2%	19.7%	46.6%	24.6%	2,237	51,059
**2010_2**	0.4%	0.1%	45.5%	20.5%	46.0%	24.8%	2,250	52,614
**2010_3**	0.5%	0.0%	46.6%	20.8%	44.5%	24.6%	2,243	52,750
**2010_4**	0.8%	0.0%	48.3%	21.1%	48.2%	24.6%	2,105	52,064
**2011_1**	0.6%	0.0%	46.7%	20.8%	48.6%	23.5%	2,021	51,771
**Intercept**	0.5%	0.3%	31.6%	20.7%	33.8%	34.2%	–	–
**Slope**	-0.01%	-0.01%	0.32%	0.11%	0.31%	-0.33%	–	–

*excluding comatose.

**excluding comatose and those with 0’s.

The final analyses examine assessment practice patterns by comparing the time between the assessment reference date (marking the day used as the clinical anchor point for the RAI 2.0 assessment) and the date the assessment is signed off as complete by the assessment coordinator. Table 11 shows the annual rates for dates where this difference is less than 0 days (indicating a coding error in the date variables), 0–6 days, 7–30 days, and more than 30 days. The preferred practice pattern is for the assessment to be signed off as complete as close to the assessment reference date as possible. However, given that some team members may be submitting their minutes of service delivery as a batch for a group of patients (e.g., rehabilitation therapy minutes) some time lag is acceptable between these dates. In all years, the majority of assessments were signed off as complete within 6 days of the assessment reference date in CCC; however, only about half of LTC met this standard. The gap was greater than 30 days in about 24% of the CCC assessments done in 1996, but this rate improved dramatically over time, with less than 10% of assessments having a gap this large in 2011. In LTC the performance in this regard was even better with less than 5% of homes having a gap of greater than 30 days in assessment completion.

**Table 11:** Time between assessment reference date and date the assessment is signed off over time, by facility type (CCC and LTC), Ontario

**Year**	**Days <0 (%)**		**0-6 days (%)**		**7-30 days (%)**		**>30 days (%)**	
	**CCC**	**LTC**	**CCC**	**LTC**	**CCC**	**LTC**	**CCC**	**LTC**
**1996**	4.0		55.6		16.3		24.1	
**1997**	4.0		65.2		16.5		14.3	
**1998**	3.0		71.3		15.9		9.8	
**1999**	2.9		72.0		14.4		10.7	
**2000**	3.0		68.1		16.0		13.0	
**2001**	2.6		70.6		15.6		11.2	
**2002**	2.5		68.3		19.1		10.1	
**2003**	3.7		66.1		18.2		12.1	
**2004**	1.0		69.1		19.9		10.1	
**2005**	0.0	0	70.6	46.13	20.3	34.42	9.1	19.46
**2006**	0.0	0	67.2	46.9	22.3	42.79	10.6	10.31
**2007**	0.0	0	64.8	52.99	24.4	41.99	10.8	5.02
**2008**	0.0	0	65.6	50.8	24.0	43.72	10.3	5.47
**2009**	0.0	0	64.7	48.95	25.3	45.79	10.1	5.26
**2010**	0.0	0	62.7	53.73	26.2	43.39	11.2	2.88
**2011**	0.0	0	60.7	52.68	29.5	44.67	9.8	2.65
**Number of assessments :**	7,798	-	302,626	424,452	88,937	361,642	51,758	36,206
**Overall%:**	1.73	0	67.08	51.62	19.71	43.98	11.47	4.4

**Figure 2:**
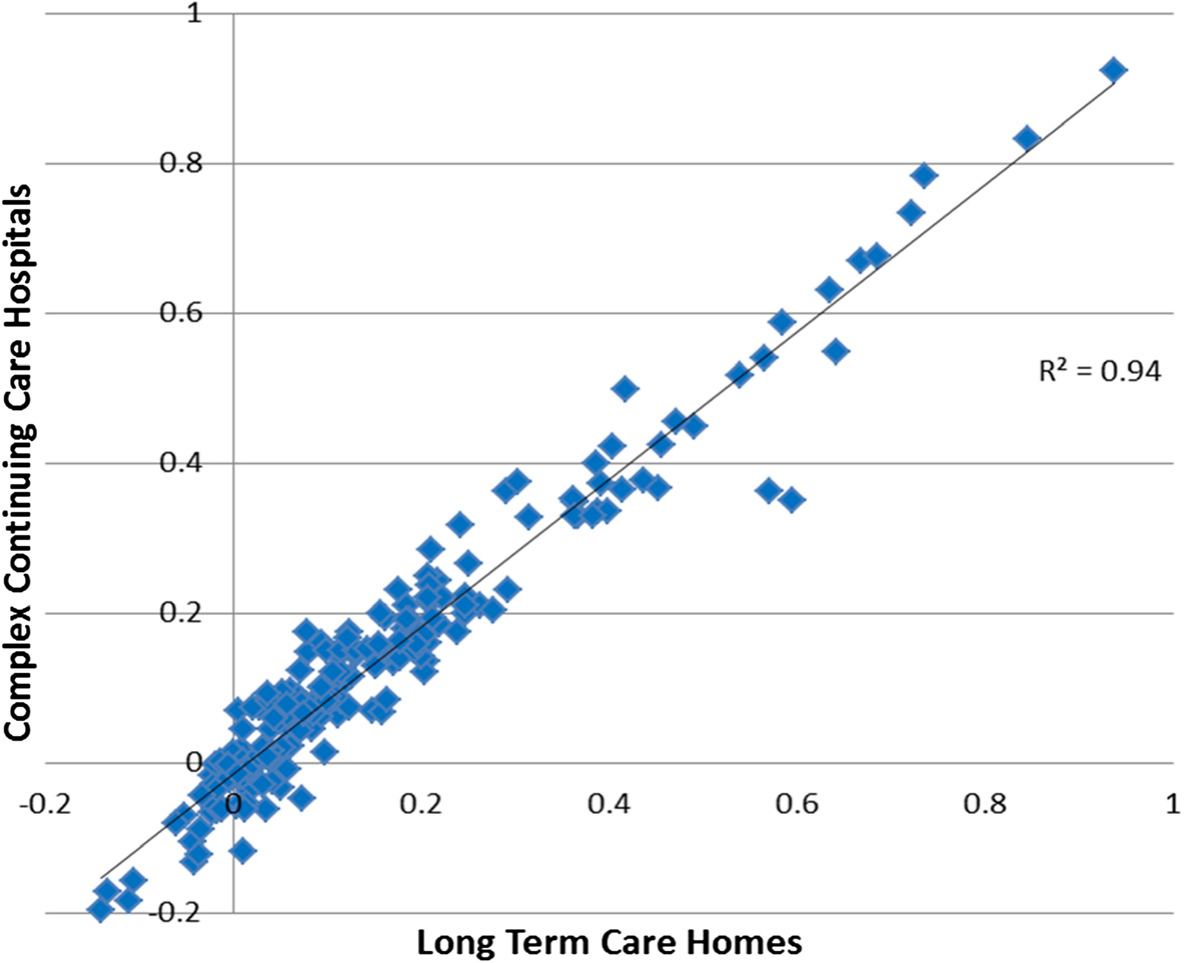
**Association between 189 statistical indicators obtained from the RAI 2.0 in CCC hospitals/units and LTC homes, Ontario, 2010.** 1 Indicators include Cronbach’s alpha values for 3 scales, 15 Pearson’s correlation coefficients among selected scales and 171 Spearman’s rank sum correlations among individual items in each setting.

Finally, Figure 2 expands on the analyses by Phillips and Morris [46] as well as those reported in Tables 4 and 5 by considering patterns of associations in clinical variables in the LTC and CCC data. The plot includes Cronbach’s alpha values for the ABS, DRS and ADL Long form scales, Pearson’s correlation coefficients for the CPS, ABS, ADL Long Form, Pain, and Changes in Health, End-Stage disease, Signs and Symptoms (CHESS) scales with each other, and Spearman’s Rank Sum correlations for 19 individual items. The result is a comparison of the associations of 185 different statistical tests between the two care settings. Figure 2 shows that there is a strong correspondence between the patterns of these associations in LTC and CCC with an R^2^ of 0.94 for the indicators between the two sectors. This suggests that the clinical elements of the RAI 2.0 behave in fundamentally the same way between the two care settings.

## Discussion

Although there are a limited number of specific findings that raise some concern, the overall picture of data quality in the CCRS between 1996 and 2011 is very positive. There is good evidence of reliability in key clinical scales. Tests of convergent validity indicate that major variables like cognition, ADL, continence, and behaviour are related in the expected directions and the associations have been stable over time. The present results replicate and extend previous analyses of US data by Phillips and Morris [46] that showed good reliability and validity in RAI 2.0 data from that country’s nursing homes. In addition, there is clear evidence that the clinical data from the RAI 2.0 behave in a consistent manner between CCC and LTC. Many other associations (e.g., cancer diagnosis and pain) were examined and yielded positive findings, but these were not reported here. Nonetheless, these relationships can be helpful as monitoring tools to examine data quality.

In addition, the rates of logical errors in clinical and service utilization indicators are low, and the efforts of CIHI to prevent such errors has proven to be effective in many areas. Although some of these errors were evident at the start of the study period, many of these rates were reduced or eliminated over time. The present findings strongly support the value of so-called “edit checks” in the CCRS to root out logical errors as noted in this report. In fact, it would be useful to extend these mechanisms to allow for longitudinal coding errors using the methods described in this paper (e.g., changes in diagnosis over time).

There is also little evidence of widespread autopopulation in the data set. To the extent that it is present, the problem is of greater concern in CCC hospitals/units than it is in LTC. Certainly, any tendency to reuse previous ratings must only be occurring at the level of limited item sets, if at all, because there is no evidence that replication of entire records occurs at more than a negligible rate. That said, it would be useful to undertake further efforts to establish the expected rate for stability in mood and ADL indicators in different settings and subpopulations. This would permit this type of check to be used as a more subtle data quality indicator than examining replication of all records. From the perspective of the participating facilities, elimination of autopopulation where it occurs should be considered a priority because it threatens the ability of the home to use interRAI Quality Indicators for quality improvement initiatives. Indeed, autopopulation will magnify the risk of a failure to detect true improvements in quality if the coding practices of the facility hide evidence of that change.

Given the notable changes in therapy minutes, nursing rehabilitation, and facilities qualifying for the Special Rehabiltation level of RUG-III, one must ask whether the change is real or the result of gaming or “upcoding” of variables to maximize case mix scores. To start, it is useful to consider what might be expected in an environment where pervasive gaming of all aspects of the RUG-III case-mix system has occurred. First, the relationship between clinical constructs (e.g., disability and cognition) would be expected to deteriorate. In fact, there is no evidence of such changes in these data. Second, the reliability of scales might be expected to decline as facilities selectively up code only those items within scales that are used to derive case-mix scores; that does not appear to have occurred in the CCRS data. There were improvements in the logical error rates for coding of therapy minutes, but the changes were modest because the rates were low at the outset.

On the other hand, for CCC hospitals/units the increased emphasis on rehabilitation and on post-acute care is entirely consistent with the policy directives of the Health Services Restructuring Commission. In addition, there was a policy initiative implemented in LTC in Ontario to expand access to rehabilitation services. This suggests that at least some of these changes were actual outcomes of policy changes in both sectors. However, it is also striking to note that a number of LTC report rehabilitation well in excess of norms in CCC hospitals/units, which requires further careful examination. A comparison with other regions that have not enacted case mix based payment may provide useful insights as to whether these changes reflect industry wide, national transitions or if they are more isolated in nature.

The present study should be extended in subsequent research to examine how the techniques reported here might be used to evaluate data quality at the facility (or even assessor) level. It is likely the case that data quality problems occur in a more pronounced way among a small number of facilities rather than in either sector as a whole. For example, a troublesome data quality finding is the tendency for a few facilities to have large gaps between the assessment reference date and the date the assessment is signed off as complete. Anecdotal reports from the field have indicated that a limited number of facilities historically relied on chart reviews to complete backlogged RAI 2.0 assessments, and this would be consistent with the findings for a handful of facilities. This practice must be strongly discouraged because it detracts from the clinical applications of the RAI 2.0. It increases the risk of not detecting major clinical problems (e.g., delirium), it excludes the patient and family from the assessment and it compromises the quality of data from the facility in question. In fact, the problem may be sufficiently important to justify rejection of assessments with a gap of greater than 30 days.

Besides the overall finding that data quality appears to be good for the CCRS, it was noteworthy that this has generally been true from the outset. Although conventional wisdom has been that the initial years of data collection for a reporting system like CCRS would yield data with compromised quality, this study provides direct evidence contrary to that assertion. While there were modest problems with coding of weight and some logical errors occurred at very low rates, the reliability and validity of the CCRS data appear to have been good from the first quarter of the mandate in each sector. While a variety of anomalies will occur with any large-scale change of this type, there is no evidence that data from the introduction of the RAI 2.0 could not have been used to inform decision-making shortly after its introduction. In fact, the primary problem with the data set in the first year was probably the absence of data from several facilities that were unable to submit data for several quarters.

The present study employs a wide set of relationships that can provide multiple perspectives from which data can be examined. This may be useful to governments, regulatory bodies, accreditation, and facility administrators who are interested in monitoring for and responding to problems in data quality. The use of statistical techniques as demonstrated here represent a lower cost option as the first line of data quality monitoring than using more expensive and burdensome techniques (e.g., widespread inter-rater reliability testing).

There are at least two ways that these analyses may be used in regular practice. First, the techniques reported here, when applied at the facility level, may be used to identify individual facilities for more careful, in-person scrutiny by expert assessors or representatives of regulatory agencies. Second, the present results may be used as benchmarks to evaluate the quality of implementation of the RAI 2.0 in other jurisdictions. For example, the effectiveness of implementing the RAI 2.0 in long term care facilities in other provinces can be examined, at least in part, by replicating the present analyses for those homes and comparing the results with the Ontario experience.

The positive findings reported here should not be taken to mean the CCRS will yield high quality data without effort. In fact, these results point to the benefits of implementing systematic checks and balances to ensuring data quality. In addition, while there are many positive findings, there remain some important areas of concern that must be addressed expeditiously. There continues to be a strong role for ongoing education and feedback to clinicians to ensure that good assessment practices are sustained over time. In that regard, the present findings provide an historical watermark of what has been achieved in Ontario. It behooves all stakeholders in the CCRS to ensure the quality of CCRS data will be sustained and improved as it becomes more widely used as a basis for decision making in clinical practice, service delivery and policy.

The present findings also point to a methodology that may be employed by other reporting systems based on newer interRAI instruments including CIHI’s Home Care Reporting System based on the RAI-Home Care [9,54] and Mental Health Reporting System for the RAI-Mental Health and interRAI Community Mental Health [55-57]. The present study was based on the RAI 2.0, which has been updated with the newer interRAI Long Term Care Facility (LTCF) instrument [8]. It will be useful to examine the performance of the various statistical indicators reported here in jurisdictions that have begun to implement the newer instrument.

An interesting question for future research is whether, as one might expect, implementations of interRAI instruments that emphasize clinical applications over their administrative uses will yield higher quality data. The present results provide a baseline of data quality measures against which alternative training and implementation approaches may be evaluated. In addition, future work might also extend these analyses by examining the extent to which reported changes in the amount of therapies (e.g., nursing rehabilitation and restorative care) actually translate to positive outcomes in the areas in which those therapies are reported to have been provided.

## Conclusions

The CCRS provides a robust, high quality data source that may be used to inform policy, clinical practice and service delivery in Ontario. The overall picture provided by these analyses provides strong evidence that the RAI 2.0 data from CCRS could appropriately be used for research use, program planning, evaluation and quality monitoring. Only one area of concern was noted (coding related to the Special Rehabilitation RUG-III level in LTC after 2009), and the statistical techniques employed here may be readily used to target organizations with data quality problems in that (or any other) area. There was also evidence that data quality was good in both sectors from the outset of implementation, meaning data may be used from the entire time series. The methods employed here may continue to be used to monitor data quality in this province over time and they provide a benchmark for comparisons with other jurisdictions implementing the RAI 2.0 in similar populations.

## Competing interests

The authors have no financial or non-financial interests to declare.

## Authors’ contributions

JPH conceived of the study and drafted the manuscript. HC, JWP, KR and NJ performed the statistical analyses. GT, JNM and BEF participated in the study design and coordination and helped to draft the manuscript. All authors read and approved the final manuscript.

## Pre-publication history

The pre-publication history for this paper can be accessed here:

http://www.biomedcentral.com/1472-6947/13/27/prepub

## References

[B1] Greiver M Barnsley J Glazier RH Harvey BJ Moineddin R Measuring data reliability for preventative services in electronic medical records BMC Health Serv Res 2012 12 116 10.1186/1472-6963-12-116 22583552 PMC3442990

[B2] Kahn MG Raebel MA Glanz JM Riedlinger K Steinder JF A pragmatic framework for single-site and multisite data quality assessment in electronic health record-based clinical research Medical Care 2012 50 Supp 21 29 10.1097/MLR.0b013e318257dd67 PMC3833692 22692254

[B3] Weiner MG Lyman JA Murphy S Weiner M Electronic health records: high-quality data for higher-quality clinical research Inform Prim Care 2007 15 2 121 127 17877874 10.14236/jhi.v15i2.650

[B4] Hirdes JP Mitchell L Maxwell CJ White N Beyond the “iron lungs of gerontology”: using evidence to shape the future of nursing homes in Canada Can J Aging 2011 30 3 371 390 10.1017/S0714980811000304 21851753

[B5] Morris JN Hawes C Fries BE Phillips CD Mor V Katz S Murphy K Drugovich ML Friedlob AS Designing the national resident assessment instrument for nursing homes Gerontologist 1990 30 3 293 302 10.1093/geront/30.3.293 2354790

[B6] Mor V A comprehensive clinical assessment tool to inform policy and practice: applications of the minimum data set Medical Care 2004 42 4 Suppl III50 III59 15026672 10.1097/01.mlr.0000120104.01232.5e

[B7] Bernabei R Gray L Hirdes J Pei X Henrard JC Jonsson PV Onder G Gambassi G Ikegami N Ranhoff AH Carpenter IG Harwood RH Fries BE Morris JN Steel K Halter JB, Ouslander JG, Tinetti ME, Studenski S, High KP, Asthana S International Gerontology in Hazzard’s Geriatric Medicine and Gerontology 6th Edition 2009 New York: McGraw Medical 69 96

[B8] Onder G Carpenter I Finne-Soveri H Gindin J Frijters D Henrard JC Nikolaus T Topinkova E Tosato M Liperoti R Landi F Bernabei R SHELTER project. Assessment of nursing home residents in Europe: the Services and Health for Elderly in Long TERm care (SHELTER) study. BMC Health Serv Res 2012 12 5 10.1186/1472-6963-12-5 22230771 PMC3286368

[B9] Gray LC Berg K Fries BE Henrard JC Hirdes JP Steel K Morris JN Sharing clinical information across care settings: the birth of an integrated assessment system BMC Health Serv Res 2009 29 9 71 19402891 10.1186/1472-6963-9-71 PMC2685125

[B10] Hirdes JP Ljunggren G Morris JN Frijters DH Finne Soveri H Gray L Björkgren M Gilgen R Reliability of the interRAI suite of assessment instruments: a 12-country study of an integrated health information system BMC Health Serv Res 2008 8 277 10.1186/1472-6963-8-277 19115991 PMC2631461

[B11] Morris JN Berg K Bjorkgren M Finne-Soveri H Fries BE Frijters D Gilgen R Gray L Hawes C Henrard JC Hirdes JP Ljunggren G Nonemaker S Steel K Szczerbinska K interRAI Clinical Assessment Protocols (CAPs) for use with Community and Long Term Care Assessment Instruments Version 9.1 2007 Rockport MA: Open Book Systems

[B12] Fries BE Morris JN Bernabei R Finne-Soveri H Hirdes J Rethinking the Resident Assessment Protocols (Letter to the Editor) J Am Geriatr Soc 2007 55 7 1139 1140 10.1111/j.1532-5415.2007.01207.x 17608893

[B13] Fries BE Schneider DP Foley WJ Gavazzi M Burke R Cornelius E Refining a case-mix measure for nursing homes: Resource Utilization Groups (RUG-III) Medical Care 1994 32 7 668 685 10.1097/00005650-199407000-00002 8028403

[B14] Mor V Improving the quality of long-term care with better information Milbank Q 2005 83 3 333 364 10.1111/j.1468-0009.2005.00405.x 16201996 PMC2690144

[B15] Zimmerman DR Karon SL Arling G Clark BR Collins T Ross R Sainfort F Development and testing of nursing home quality indicators Health Care Financ Rev 1995 16 4 107 127 10151883 PMC4193525

[B16] Doran DM Harrison MB Laschinger HS Hirdes JP Rukholm E Sidani S McGillis Hall L Tourangeau AE Nursing Sensitive Outcomes Data Collection in Acute Care and Long-Term Care Settings Nurs Res 2006 55 S75 S81 10.1097/00006199-200603001-00012 16601638

[B17] Joint Policy and Planning Committee (JPPC) Cost per Case-Mix Weighted Activity for Complex Continuing Care in Ontario 1999 Toronto: JPPC http://www.ontla.on.ca/library/repository/mon/7000/10316975.pdf

[B18] Ontario Ministry of Health and Long Term Care Long-Term Care Homes Financial Policy 2011 Toronto: Ontario Ministry of Health and Long Term Care http://www.health.gov.on.ca/en/public/programs/ltc/docs/level_care_policy.pdf

[B19] Jones RN Hirdes JP Poss JW Kelly M Berg K Fries BE Morris JN Adjustment of nursing home quality indicators BMC Health Serv Res 2010 10 96 10.1186/1472-6963-10-96 20398304 PMC2881673

[B20] Canadian Institute for Health Information Health Care in Canada: A Focus on Seniors and Aging 2011 Ottawa: CIHI

[B21] Hirdes JP Botz CA Kozak J Lepp V Identifying an appropriate case-Mix measure for chronic care: evidence from an Ontario pilot study Healthc Manage Forum 1996 9 40 46 10.1016/S0840-4704(10)60943-X 10157047

[B22] Hirdes JP Long term care funding in Canada: A policy mosaic J Aging Soc Policy 2001 13 2–3 69 81 12216363 10.1300/j031v13n02_06

[B23] Hirdes JP Sinclair DG King J McKinley J Tuttle P Fries BE, Fahey CJ From anecdotes to evidence: complex continuing care at the dawn of the information Age Implementing the Resident Assessment Instrument: Case Studies of Policymaking for Long-Term Care in Eight Countries 2003 New York: Milbank Memorial Fund Published as a peer-reviewed, electronic document at http://www.milbank.org/reports/interRAI/030222interRAI.html

[B24] Hirdes JP Addressing the health needs of frail elderly people: Ontario’s experience with an integrated health information system Age Ageing 2006 35 4 329 331 10.1093/ageing/afl036 16788076

[B25] Teare G Hirdes JP Ziraldo M Proctor W Nenadovic M Provincial Status Report – The Quality of Caring – Ontario April 1998–1999 2000 Toronto: Canadian Institute of Health Information

[B26] Teare GF Daniel I Markel F Hospital Report 2001: Complex Continuing Care. A Joint Initiative of the Ontario Hospital Association and the Government of Ontario 2001 Toronto: Hospital Report Research Collaborative, University of Toronto

[B27] Teare GF Daniel I Markel F Hospital Report 2003: Complex Continuing Care. A Joint Initiative of the Ontario Hospital Association and the Government of Ontario 2004 Toronto: Hospital Report Research Collaborative, University of Toronto

[B28] Ontario Ministry of Health and Long Term Care Quality of Care – Quality of Life: Long-Term Care Homes Common Assessment Project Final Report 2011 Toronto: Community Care Information Management http://www.ccim.on.ca/LTCH/RAI/Document/LTCH_CAP%20Final_Report_20110112_v%201%201.pdf

[B29] Canadian Institute for Health Information Continuing Care Reporting System (CCRS) 2004–2005 Data Quality Documentation 2005 Ottawa: CIHI

[B30] Anderson RL Buckwalter KC Buchanan RJ Maas ML Imhof SL Validity and reliability of the Minimum Data Set Depression Rating Scale (MDSDRS) for older adults in nursing homes Age Ageing 2003 32 4 435 438 10.1093/ageing/32.4.435 12851189

[B31] Schnelle JF Wood S Schnelle ER Simmons SF Measurement sensitivity and the minimum data Set depression quality indicator Gerontologist 2001 41 3 401 405 10.1093/geront/41.3.401 11405438

[B32] Teresi J Holmes D Should MDS data be used for research? Gerontologist 1992 32 2 148 151 10.1093/geront/32.2.148 1577305

[B33] Hawes C Morris JN Phillips CD Mor V Fries BE Nonemaker S Reliability estimates for the minimum data Set for nursing home resident assessment and care screening (MDS) Gerontologist 1995 35 2 172 178 10.1093/geront/35.2.172 7750773

[B34] Mor V Angelelli J Jones R Roy J Moore T Morris J Inter-rater reliability of nursing home quality indicators in the U.S BMC Health Serv Res 2003 20 1 13 10.1186/1472-6963-3-20 PMC280691 14596684

[B35] Morris J Nonemaker S Murphy K Hawes C Fries BE Mor V A commitment to change: revision of HCFA’s RAI J Am Geriatric Soc 1997 45 8 1011 1016 10.1111/j.1532-5415.1997.tb02974.x 9256856

[B36] Sgadari A Morris JN Fries BE Ljunggren G Jonsson PV DuPaquier JN Schroll M Efforts to establish the reliability of the resident assessment instrument Age Ageing 1997 26 Suppl 2 27 30 10.1093/ageing/26.suppl_2.27 9464551

[B37] Bjorkgren MA Hakkinen U Finne-Soveri UH Fries BE Validity and Reliability of Resource Utilization Groups (RUG-Ill) in Finnish Long-term Care Facilities Scand J Public Health 1999 27 3 228 234 10482083 10.1177/14034948990270030201

[B38] Gambassi G Landi F Peng L Brostrup-Jensen C Calore K Hiris J Lipsitz L Mor V Bernabei R Validity of Diagnostic and Drug Data in Standardized Nursing Home Resident Assessments. Potential for Geriatric Pharmacoepidemiology Medical Care 1998 36 2 167 179 10.1097/00005650-199802000-00006 9475471

[B39] Hawes C Phillips CD Mor V Fries BE Morris JN MDS data should be used for research Gerontologist 1992 32 4 563 564 1427264 10.1093/geront/32.4.563b

[B40] Hjaltadóttir I Rahm Hallberg I Kristensson E Nyberg P Predicting mortality of residents at admission to nursing home: A longitudinal cohort study BMC Health Serv Res 2011 11 86 10.1186/1472-6963-11-86 21507213 PMC3112069

[B41] Koehler M Rabinowitz T Hirdes J Stones M Carpenter IG Fries BE Morris JN Jones RN Measuring depression in nursing home residents with the MDS and GDS: an observational psychometric study BMC Geriatr 2005 5 1 10.1186/1471-2318-5-1 15627403 PMC546185

[B42] Martin L Poss JW Hirdes JP Jones RN Stones MJ Fries BE Rabinowitz T Predictors of a new depression diagnosis among older adults admitted to complex continuing care: implications for the depression rating scale (DRS) Age and Aging 2008 37 1 51 56 10.1093/ageing/afm162 18033777

[B43] Mor V Intrator O Unruh MA Cai S Temporal and geographic variation in the validity and internal consistency of the nursing home resident assessment minimum data Set 2.0 BMC Health Serv Res 2011 11 78 10.1186/1472-6963-11-78 21496257 PMC3097253

[B44] Morris JN Jones R Fries BE Hirdes JP Convergent Validity of Minimum Data Set–Based Performance Quality Indicators in Postacute Care Settings Am J Med Qual 2004 19 6 242 247 10.1177/106286060401900603 15620075

[B45] Poss JW Jutan NM Hirdes JP Fries BE Morris JN Teare GF Reidel K A review of evidence on the reliability and validity of minimum data Set data Healthc Manage Forum 2008 21 1 33 39 10.1016/S0840-4704(10)60127-5 18814426

[B46] Phillips CD Morris JN The potential for using administrative and clinical data to analyze outcomes for the cognitively impaired: an assessment of the minimum data set for nursing homes Alzheimer Dis Assoc Disord 1997 11 Suppl 6 162 167 9437461

[B47] Crooks VC Schnelle JF Ouslander JP McNees MP Use of the Minimum Data Set to rate incontinence severity J Am Geriatr Soc 1995 43 12 1363 1369 7490387 10.1111/j.1532-5415.1995.tb06615.x

[B48] Hirdes JP Riddell M Curtin-Telegdi N Exchanging MDS Data Across Sectors of the Health Care System: Clinical Considerations 2001: Senior Care Canada http://seniorcarecanada.com/articles/2001/q3/exchanging.mds.data/

[B49] Morris JN Fries BE Morris SA Scaling ADLs within the MDS J Gerontology: Med Sci 1999 54A 11 M546 M553 10.1093/gerona/54.11.m546 10619316

[B50] Burrows AB Morris JN Simon SE Hirdes JP Phillips C Development of a Minimum Data Set-based depression rating scale for use in nursing homes Age and Aging 2000 29 2 165 172 10.1093/ageing/29.2.165 10791452

[B51] Perlman CM Hirdes JP The aggressive behavior scale: a New scale to measure aggression based on the minimum data Set J Am Geriatr Soc 2008 56 12 2298 2303 10.1111/j.1532-5415.2008.02048.x 19093929

[B52] Morris JN Fries BE Mehr DR Hawes C Phillips C Mor V Lipsitz LA MDS Cognitive Performance Scale J Gerontology: Med Sci 1994 49 4 M174 M182 10.1093/geronj/49.4.M174 8014392

[B53] Proctor WR Hirdes JP Pain and cognitive status among nursing home residents in Canada J Pain Res Manage 2001 6 3 119 125 10.1155/2001/978130 11854774

[B54] Morris JN Fries BE Steel K Ikegami N Bernabei R Carpenter GI Gilgen R Hirdes JP Topinková E Comprehensive clinical assessment in community setting: applicability of the MDS-HC J Am Geriatr Soc 1997 45 8 1017 1024 9256857 10.1111/j.1532-5415.1997.tb02975.x

[B55] Hirdes JP Smith TF Rabinowitz T Yamauchi K Pérez E Telegdi NC Prendergast P Morris JN Ikegami N Phillips CD Fries BE Resident Assessment Instrument-Mental Health (RAI-MH): inter-rater reliability and convergent validity J Behav Health Serv Res 2002 29 4 419 432 10.1007/BF02287348 12404936

[B56] Mathias K Hirdes JP Pittman D A care planning strategy for traumatic life events in community mental health and inpatient psychiatry based on the InterRAI assessment instruments Comm Ment Health J 2010 46 6 621 627 10.1007/s10597-010-9308-2 20449657

[B57] Perlman CM Hirdes JP Barbaree H Fries BE McKillop I Morris JN Rabinowitz T Development of mental health quality indicators (MHQIs) for inpatient psychiatry based on the interRAI mental health assessment BMC Health Serv Res 2013 13 15 10.1186/1472-6963-13-15 23305286 PMC3560122

